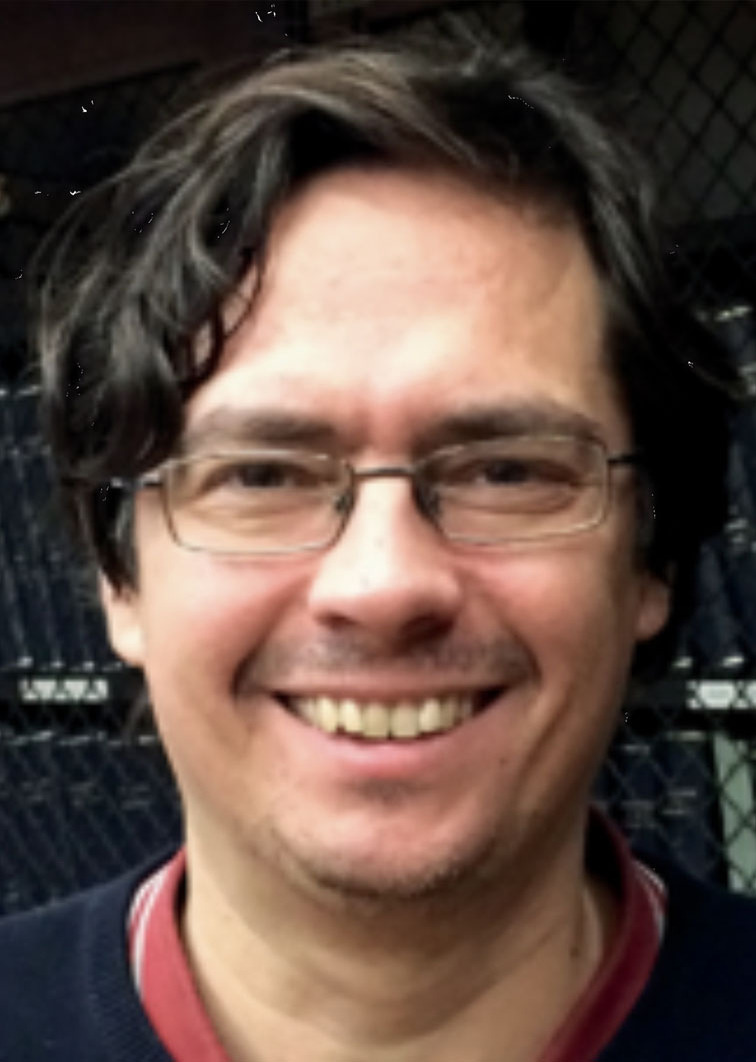# Chemically Fueled Self‐Assembly in Biology and Chemistry

**DOI:** 10.1002/anie.202100274

**Published:** 2021-04-07

**Authors:** Krishnendu Das, Luca Gabrielli, Leonard J. Prins

**Affiliations:** ^1^ Department of Chemical Sciences| University of Padova Via Marzolo 1 35131 Padova Italy

**Keywords:** chemical fuel, non-equilibrium systems, origin of life, self-assembly, systems chemistry

## Abstract

Life is a non‐equilibrium state of matter maintained at the expense of energy. Nature uses predominantly chemical energy stored in thermodynamically activated, but kinetically stable, molecules. These high‐energy molecules are exploited for the synthesis of other biomolecules, for the activation of biological machinery such as pumps and motors, and for the maintenance of structural order. Knowledge of how chemical energy is transferred to biochemical processes is essential for the development of artificial systems with life‐like processes. Here, we discuss how chemical energy can be used to control the structural organization of organic molecules. Four different strategies have been identified according to a distinguishable physical‐organic basis. For each class, one example from biology and one from chemistry are discussed in detail to illustrate the practical implementation of each concept and the distinct opportunities they offer. Specific attention is paid to the discussion of chemically fueled non‐equilibrium self‐assembly. We discuss the meaning of non‐equilibrium self‐assembly, its kinetic origin, and strategies to develop synthetic non‐equilibrium systems.

## Graphical abstract

Life is a non‐equilibrium state of matter maintained at the expense of energy. Nature uses chemical energy stored in molecules to sustain non‐equilibrium structures. This Review describes how chemical energy can be used to control the self‐assembly of organic molecules. The ability of chemists to implement the described strategies in a synthetic context will permit the development of artificial systems with life‐like properties.

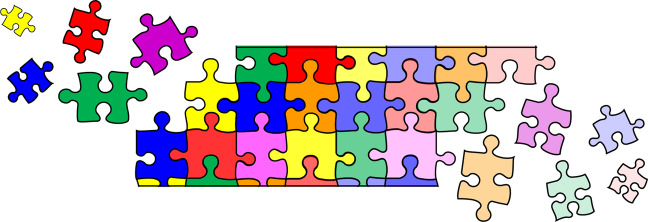

## 1 Introduction

The question of how life originated on earth touches the essence of humankind and has led to profound existential reflections by theologists, philosophers, and scientists over the course of human history. Scientists marvel at the observation that living matter is able to defy the second law of thermodynamics by maintaining local order in a universal context of a continuous increase in entropy.[^1^, ^2^, ^3^] This astonishing feature implies that the acquisition of the ability to exploit energy from the surroundings to create and maintain structure must have played a key part in the transition from inert to living matter.[^4^, ^5^] Energy is abundantly available on earth in the form of photons that are generated by nuclear fusion processes occurring in the sun and in the form of geothermal energy originating from the earth's core. Although other energy sources are available, the importance of solar and geothermal energy is that they can directly activate molecules and facilitate chemical reactions. The photo‐ or thermal activation of molecules enables their conversion into new molecules with increased chemical potential.^[6]^ The storage of solar energy in high‐energy molecules through photosynthesis is the process that sustains life on earth.[^7^, ^8^] The chemical energy stored in thermodynamically activated but kinetically stable molecules drives the entire biological machinery.^[9]^

The non‐equilibrium nature of life is manifest in the cell, the smallest organizational unit that expresses the characteristics of life.^[10]^ Cells grow and divide, reproduce, communicate with the environment, and adapt to changes therein.^[11]^ The expression of these features requires a continuous supply of energy, which arrives at the cell in the form of high‐energy nutrients; death follows when the energy supply ceases. The energy released from the breakdown of nutrients is used for the synthesis of lipids, peptides, and nucleic acids, which self‐assemble in a thermodynamically controlled manner to form the constitutional structures of the cell, including membranes, proteins, and the genome. Importantly, energy is also used to synthesize molecules with a high chemical potential—for example, ATP, NADH, acetyl‐CoA—which serve as chemical fuels that keep the biological engine running.^[9]^ The energy released from these molecules upon their conversion into waste molecules with a lower chemical potential drives molecular pumps and motors, which results in sustained concentration gradients and directional motion; these are evident signatures of a non‐equilibrium system.[^12^, ^13^, ^14^, ^15^] At the same time, chemical fuels also play a direct role in the structural organization of the cell by controlling self‐assembly processes in time and space.[^16^, ^17^, ^18^] Through a direct coupling of self‐assembly processes and energy dissipation processes, chemical fuels also allow the formation of high‐energy structures, which is another signature of the non‐equilibrium nature of life.^[19]^

Driven by the desire to understand how life could have originated in a prebiotic context, chemists have had a longstanding interest in the origin‐of‐life question.[^20^, ^21^, ^22^, ^23^, ^24^, ^25^, ^26^, ^27^] In more recent years, interest in the chemistry of life has gained even more traction, as chemistry has advanced to such a level that the installation of emergent properties in complex synthetic mixtures of interacting molecules, just as in the cell, has become within experimental reach from a synthetic and analytical point of view.[^28^, ^29^, ^30^] The implementation of life‐like properties in synthetic systems offers the prospective of unprecedented materials and, in the future, the development of artificial entities that may be recognized as being alive.^[31]^ In these studies, the development of synthetic chemical systems that operate out‐of‐equilibrium form a central focus.[^32^, ^33^, ^34^, ^35^] Great strides have been made in the design of artificial molecular machines that exploit energy to function.[^36^, ^37^, ^38^, ^39^, ^40^, ^41^, ^42^, ^43^, ^44^, ^45^, ^46^, ^47^] Although light has most frequently been used as an energy source, the first molecular machines that exploit chemical energy have also been recently reported.[^48^, ^49^, ^50^, ^51^, ^52^] Compared to molecular machines, the self‐assembly of non‐equilibrium structures is still in its infancy.[^53^, ^54^, ^55^, ^56^, ^57^, ^58^, ^59^, ^60^, ^61^] Although highly interesting systems with novel properties have been described, progress towards systems that can mimic natural non‐equilibrium self‐assembly in all its facets could be improved by a better understanding of the physical‐chemical principles that govern the transfer of energy from a chemical fuel to the self‐assembly process.[^62^, ^63^]

In this Review, we discuss in a systematic manner how chemical fuels can be used to control the self‐assembly of organic molecules. The objective of this Review is to provide a systematic, accessible treatment that extends on the more technical analysis that we and others have reported previously on the topic of chemically fueled non‐equilibrium self‐assembly.[^62^, ^63^, ^64^] We have identified four different classes that differ in the way chemical energy stored in molecules is transferred to the self‐assembly process. Class 1 describes the use of templates to control self‐assembly processes at thermodynamic equilibrium. Class 2 describes templated self‐assembly under dissipative conditions, which implies that the template is gradually converted into a nontemplating waste molecule by an external agent that does not participate in the self‐assembly process. Class 3 describes systems in which the self‐assembling components play an active role in the conversion of fuel into waste. Class 4 describes systems similar to Class 3 but with the additional feature that the released chemical energy is used to drive the system out‐of‐equilibrium.

These classes will be presented in order of increasing complexity and for each of them a conceptual analysis will be provided followed by two representative examples: one from biology and one from chemistry. The reason for this setup is that the illustration of the concept with practical examples shows how an abstract concept can be translated to molecular systems. The direct comparison of examples from nature and the laboratory provides insight into how the same concept can be applied to chemical systems with very different levels of complexity. We would like to point out that this Review is not intended to be comprehensive; key examples were selected from the rich literature based on their suitability to illustrate the concept. Excellent and comprehensive reviews of the respective research areas that will be discussed herein are available in the literature and references to these have been inserted to guide the reader.

**Figure 1:**
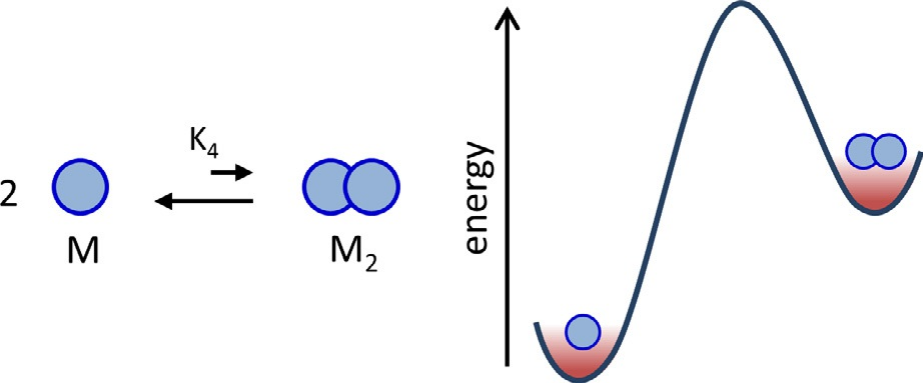
The equilibrium between M and M_2_ that is used as a reference throughout the Review. The composition at equilibrium is defined by the equilibrium constant *K*_4_ (m ^−1^). The notation for the equilibrium constant (*K*_4_) is used to facilitate the comparison of the discussion in this Review with a previous publication.^[62]^

To facilitate the analysis and comparison between the different classes, we have chosen the self‐assembly of two identical monomers (M) into dimer M_2_ as the framework for the conceptual analysis (Figure 1). Dimerization represents the simplest example of the organization of molecules. The composition at thermodynamic equilibrium is defined by an equilibrium constant, *K*_4_, which in our analysis is chosen such that the equilibrium composition resides on the side of the monomers. This implies that dimer M_2_ is higher in energy than monomer M and that self‐assembly does not occur spontaneously.

## 2 Class 1: Templated Self‐Assembly

### 2.1 Concept

**Figure 2:**
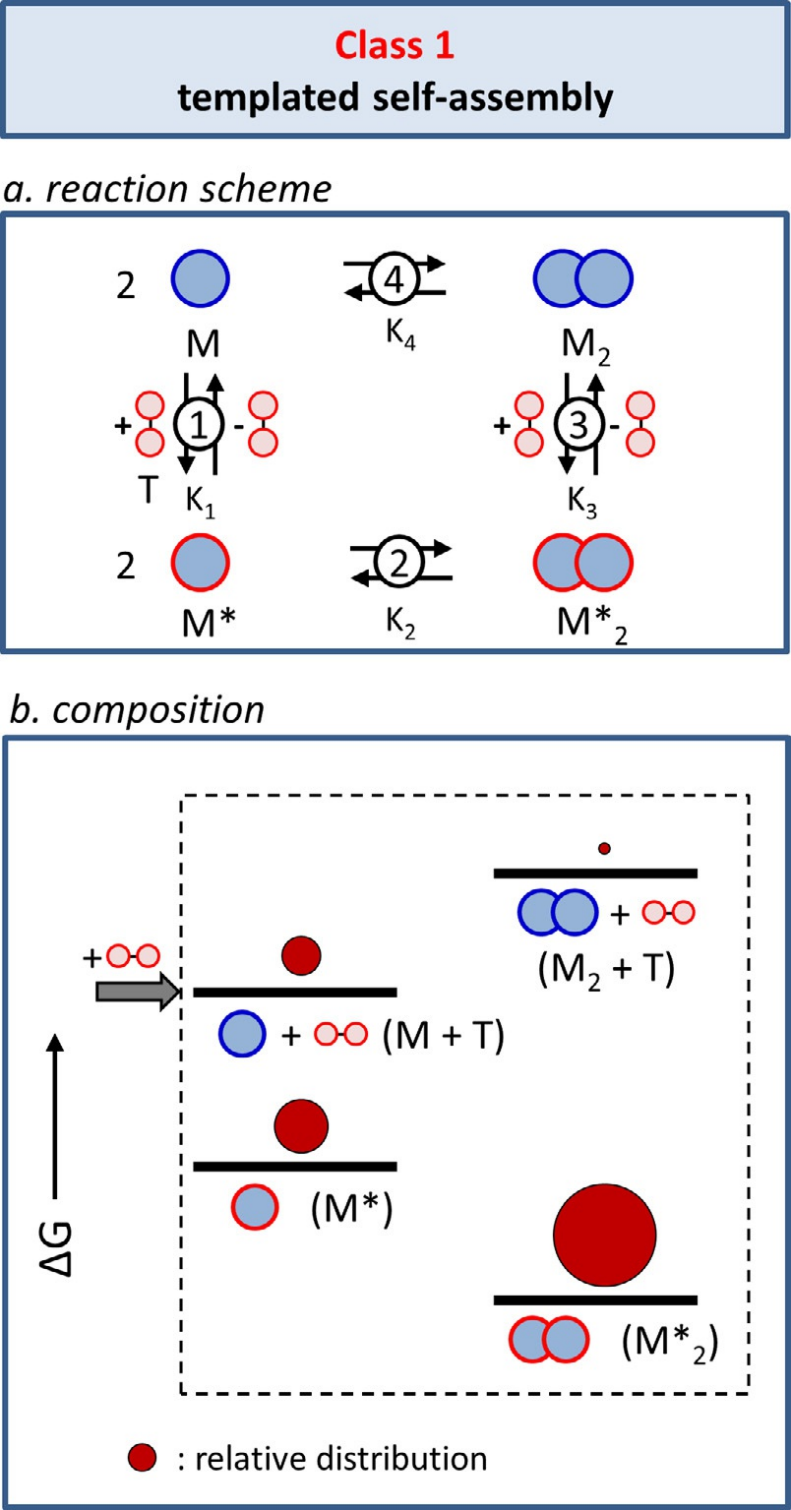
Class 1: Templated self‐assembly. a) The reaction scheme describes the chemical connectivity between the components of the system. b) Energy diagram in which the dark red circles indicate the composition of the system in the presence of template T.

An evaluation of templated self‐assembly forms the best starting point for our analysis, even though no energy is dissipated by the chemical conversion of fuel into waste. Templated self‐assembly sets the proper framework for the analysis of the other classes, in which energy dissipation does take place. The distribution of species under equilibrium conditions is defined by the thermodynamic cycle depicted in Figure 2 a. The interaction of template T with M leads to a complex M*, which is activated for dimerization to give the thermodynamically more stable dimer M*_2_. Dissociation of the template from M*_2_ results in the formation of the high‐energy species M_2_, which dissociates to give M. The most populated species at equilibrium is M*_2_, which has the highest thermodynamic stability.

Microscopic reversibility implies that the reaction path in the reverse direction must in every detail be the identical reverse of the reaction path in the forward direction.[^65^, ^66^] To emphasize this aspect, in this Review we consistently describe all reactions as equilibria, even those that, from a practical point of view, would typically be considered unidirectional. At the molecular level, microscopic reversibility leads to the concept of detailed balance, which states that in a system at thermodynamic equilibrium the rate of the forward reaction must be identical to the rate of the backward reaction. A consequence of microscopic reversibility for the thermodynamic cycle depicted in Figure 2 a is Equation 1.

(1) $${K_{1}^{2}K}_{2}K_{3}^{-1}K_{4}^{-1}=1$$

This equation implies that, from an energetic point of view, there is no difference if the activated assembly M*_2_ is populated by dimerization of M followed by association of the template or by activation of M followed by the dimerization of M*. In other words, from an energetic point of view, there is no difference in following a thermodynamic cycle in the clockwise or counterclockwise direction.

For all the different classes of fueled self‐assembly that will be discussed we have added energy diagrams containing the relative energy levels of the different states. These schemes serve to facilitate an understanding of how the population of the states is affected by the addition of fuel/template and the conversion of fuel into waste (for the successive classes). For Class 1, this results in a four‐state diagram (Figure 2 b) with the distribution of M amongst the four states represented by the size of the dark red circles. The reported (arbitrary) distribution is based on simulations reported previously.^[62]^ From the scheme it is evident that the distribution is completely dictated by the relative thermodynamic stabilities.

### 2.2 Biology—Templated Self‐Assembly of Virus Capsids

Self‐assembly is extensively used in nature for the structural organization of matter. The formation of self‐assembled structures that need to persist in time typically involve building blocks (phospholipids, nucleic acids, peptides) that spontaneously assemble through a thermodynamically controlled process. Nature makes such building blocks by exploiting the energy released from the degradation of nutrients in catabolic pathways. However, on certain occasions the use of templates in the self‐assembly of thermodynamically stable structures provides additional benefits, which is illustrated by taking the self‐assembly of virus capsids templated by nucleic acids as an example.

Viruses are dynamic assemblies of nucleic acids and proteins that use their own components and the biological machinery of a cell to multiply and propagate.^[67]^ Virions—single infectious viral particles—exist with various sizes, shapes, and levels of complexity.^[68]^ Virions range from very simple structures composed of just the viral nucleic acid and a surrounding protein shell, called a capsid, to complex enveloped structures in which a lipid membrane surrounds the capsid and which contain additional proteins both inside the capsid and expressed on the envelope.^[69]^ Capsid formation is a beautiful example of a self‐assembly process; hundreds of copies of a capsid protein spontaneously assemble to form an object that can reach even micrometer‐sized dimensions.^[70]^ Generally, viral capsids have either a rodlike shape, in which the capsid proteins are arranged in a helical fashion around the nucleic acid, or a spherical shape, in which the capsid proteins are arranged with icosahedral symmetry. Since a helix is, in principle, an infinite structure, rod‐shaped capsids can, in principle, accommodate nucleic acids of any length. On the other hand, the geometrical constraints of the icosahedral lattice require the spherical capsids to be composed of 60*T* subunits, in which *T* is the triangulation number, which is equal to the number of distinct conformations of the capsid protein that can be found in the lattice.

Virions can form as empty capsids, so‐called procapsids, which are successively loaded with nucleic acids through a portal protein complex using an ATP‐driven molecular pump. This is typically the case for double‐stranded DNA (dsDNA) or dsRNA viruses, which have such a high stiffness and charge density that spontaneous encapsulation is precluded. However, here we want to focus on those virions that form through a process in which the nucleic acid, typically a flexible single nucleic acid strand (mostly ssRNA), serves as the template for the self‐assembly of the capsid and discuss the advantages of templation.[^71^, ^72^]

**Figure 3:**
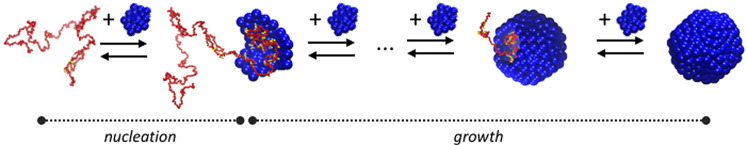
Schematic representation of the templated self‐assembly of a virion following a nucleation‐growth model. Figure inspired by Ref. [73].

One of the mechanisms for the templated self‐assembly of capsids is a nucleation‐and‐growth sequence, in which a small cluster of capsid proteins engages in interactions with ssRNA (Figure 3).^[73]^ Electrostatic interactions between the negatively charged nucleic acid strand and the flexible N‐terminal domain of a capsid protein rich in basic amino acids—called arginine‐rich motifs (ARMs)—form the thermodynamic driving force for the formation of the nucleation complex. The favorable contribution of the ssRNA to the formation of this complex allows self‐assembly of the nucleation complex to occur at lower concentrations compared to the threshold concentration required for empty capsid assembly (*c*_templated_<*c*_empty_). Formation of the nucleation complex frequently involves specific short RNA sequences of the nucleic acid strand with a well‐defined secondary structure, so‐called packaging signals. Nucleation is followed by the condensation of additional capsid proteins. In this growth step, interactions between RNA and the capsid protein serve not only to promote oligomerization, but also to direct the self‐assembly pathway. Both specific and nonspecific interactions lead to the simultaneous packaging of the nucleic acid strand and conformational changes in the capsid protein required to form a stable assembly. The selectivity of the interaction between the nucleic acid strand and capsid proteins in this templated self‐assembly process is part of the reason why virions can assemble with specificities up to 99 % around the viral genome in a cellular context that contains a myriad of nonviral RNA molecules.

### 2.3 Chemistry—Dynamic Combinatorial Chemistry

**Figure 4:**
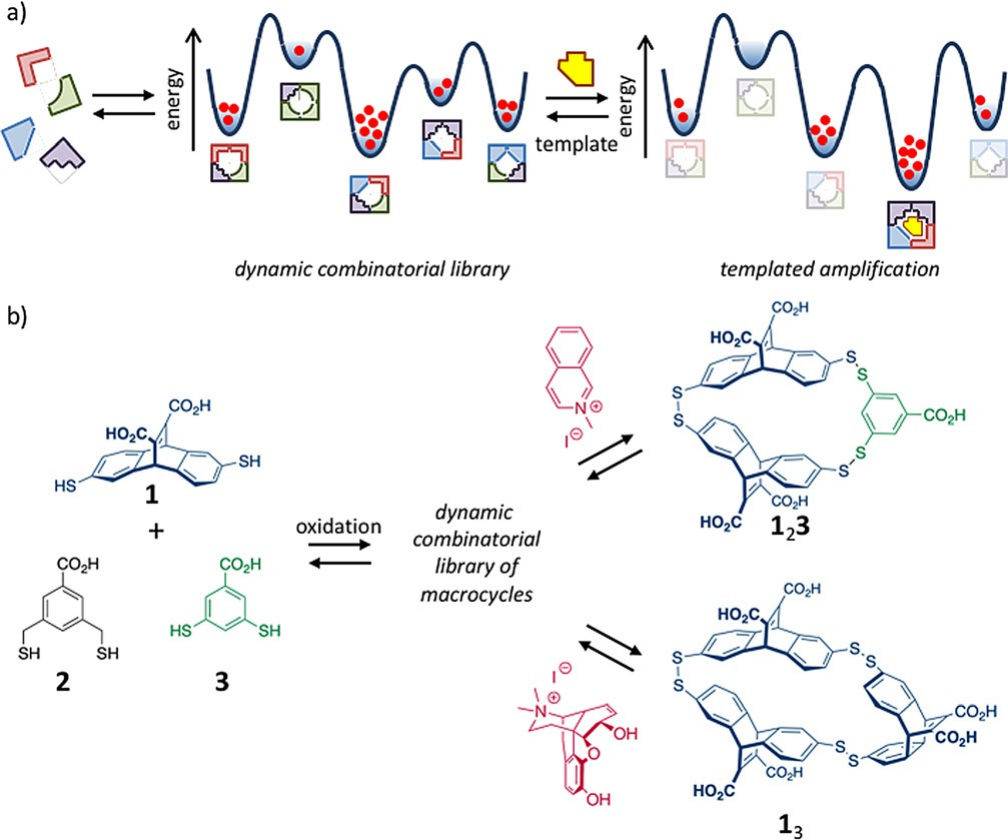
a) Schematic representation of the formation of a dynamic combinatorial library from a mixture of building blocks and the spontaneous adaptation to the addition of a template. b) Mixing building blocks **1**–**3** results in the formation of a dynamic library of macrocycles containing at least 45 distinct structures. The addition of templates causes selective amplification of library members.^[85]^

The serendipitous discovery by Pedersen that Na^+^ cations templated the formation of crown ethers initiated the era of supramolecular chemistry.[^74^, ^75^, ^76^] Since its inception, the use of templates to control self‐assembly processes has maintained a prominent position in supramolecular chemistry, eventually culminating in the development of dynamic combinatorial chemistry (DCC) as a tool for the discovery of receptors, catalysts, and materials (Figure 4 a).[^77^, ^78^, ^79^, ^80^, ^81^, ^82^, ^83^, ^84^] DCC relies on the use of dynamic combinatorial libraries, which are composed of dynamic members that can interconvert because the building blocks interact through reversible bonds (either noncovalent or covalent). These libraries are at thermodynamic equilibrium, which implies that the library composition is dictated by the relative stability of each library member in the global energy landscape. The central concept of DCC is the adaptation of the system to a perturbation in the energy landscape caused by an external stimulus. For example, the addition of a templating molecule will spontaneously lead to a change in the library composition, enriched in the library member with, in principle, the highest affinity for the template. That library member can be identified in a straightforward manner simply by comparing the library compositions before and after addition of the stimulus. The attractiveness of DCC is that screening is carried out by the molecules themselves, which overcomes the limitations of rational design.

The potential of DCC has been nicely illustrated by Otto et al., who demonstrated that a single dynamic combinatorial library can evolve in different directions depending on the molecular structure of the added template (Figure 4 b).^[85]^ A dynamic library was obtained by mixing three different dithiol building blocks **1**–**3**, which under slowly oxidizing conditions formed a myriad of disulfide macrocycles of different sizes and composition. ESI‐MS measurements of the library revealed the presence of 45 library members with a distinct mass. Exposure of the library to 2‐methylisoquinolinium iodide resulted in a major change in the library composition, as observed by HPLC analysis. A strong amplification was observed for macrocycle **1**_2_ **3**, which was a library member hardly detectable in the template‐free library. Notably, exposure of the same library to a different template—N‐methylated morphine—led to strong amplification of a completely different macrocycle, namely **1**_3_. It is worth noticing that the template‐induced increase in macrocycles enriched in building block **1** is accompanied by a decrease in other macrocycles containing **1**, which illustrates that the amplification process indeed occurs at the expense of competing library members. Respective binding constants of 2.5×10^5^ m ^−1^ and 7.1×10^5^ m ^−1^ were determined for complexes between macrocycles **1**_2_ **3** and **1**_3_ and their respective guests, which illustrate that DCC permits high‐affinity receptors to be identified from a mixture of structurally related structures.

## 3 Class 2: Templated Self‐Assembly under Dissipative Conditions

### 3.1 Concept

Templates favor self‐assembly processes because the interaction with the self‐assembling components is an energetically downhill process. In the example of virion self‐assembly, we have seen that the association of capsid proteins to the nucleic acid strand increases their local concentration, which permits self‐assembly to occur at lower concentrations. There is a clear entropic contribution to this process which results from the exchange of multiple counteranions from the positively charged amino acid residues in the capsids by a single one—the nucleic acid strand. It is important to note that the biochemical synthesis of such a single multicharged counteranion is an energetically uphill process. From this analysis it follows that templates of this kind can be regarded as high‐energy molecules. In principle, this energy can be released through an independent chemical reaction that breaks down the template, which consequently loses its templating ability. This situation defines Class 2: Templated self‐assembly under dissipative conditions.

**Figure 5:**
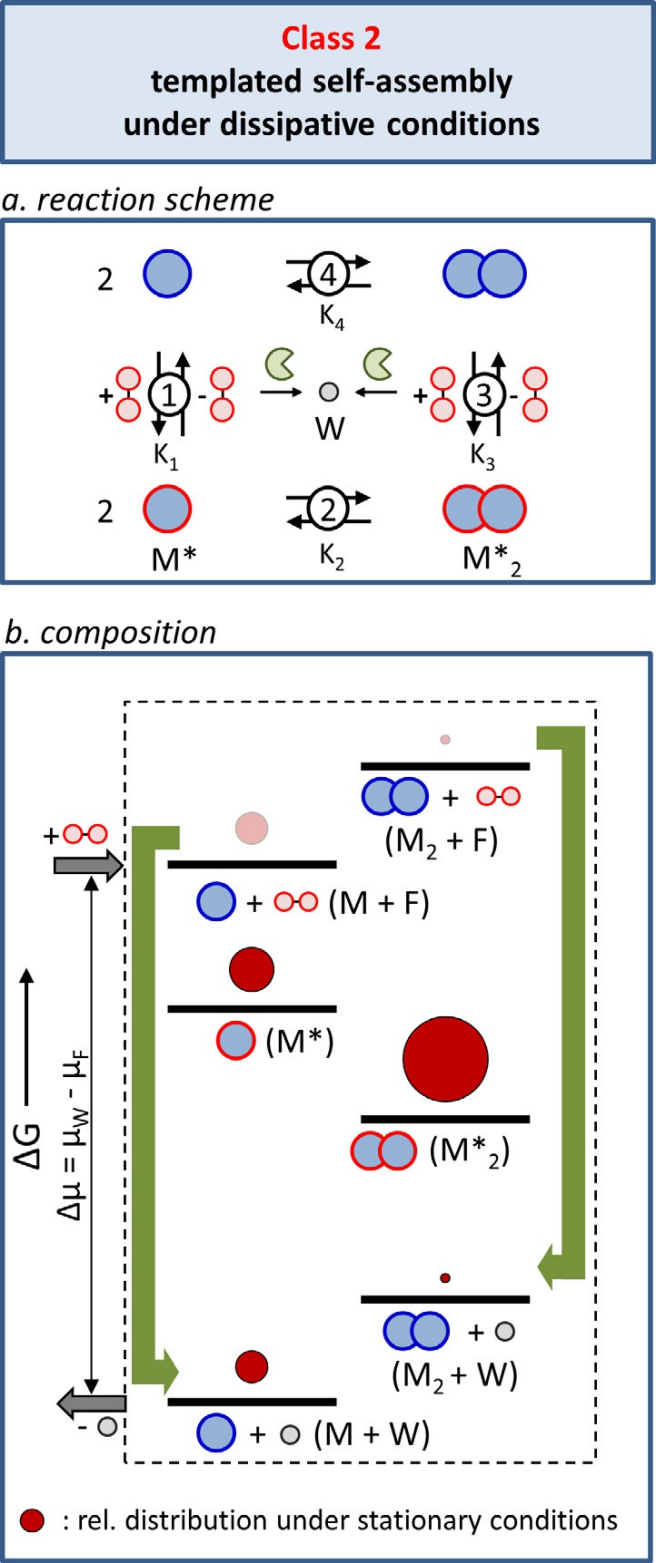
Class 2: Templated self‐assembly under dissipative conditions. a) The reaction scheme describes the chemical connectivity between the components of the system and the energy dissipation process. b) Energy diagram illustrating the composition of the system under stationary conditions, that is, at constant fuel and waste concentrations in an open system. The green arrows indicate the energy dissipation steps. The size of the dark red circles indicates the relative populations of the respective states reported in previous simulations.^[62]^

A system displaying templated self‐assembly under dissipative conditions contains the identical set of equilibria as Class 1 (Figure 5 a). The difference lies in the fact that an additional process is present that slowly degrades the template into a waste product (W) without templating ability. The degradation process is caused by an external element (for example, an enzyme or reactant) or by the experimental conditions; the monomers and assemblies do not play a role in this process. An important consequence of template degradation is that, just as in Cass 1, after the initial change in the energy landscape upon the addition of a batch of template, the energy landscape gradually reverts to the original energy landscape (assuming that the waste molecules do not interfere in the self‐assembly process).

This difference with Class 1 is represented in the energy diagram by the presence of two novel energy states corresponding to M+W and M_2_+W (Figure 5 b). These states are lower in energy compared to all other states—which involve intact template—indicative of the lower chemical potential of the waste compared to the template. The difference in the chemical potential between the fuel and waste corresponds to the energy difference between states M+F and M+W in the diagram (or between M_2_+F and M_2_+W). It should be noted that in the scheme we have changed the label for the chemical trigger from T (template) to F (fuel). It is worth spending a few words on the term “fuel”, which in the literature on chemically fueled processes is used with ambiguity.[^41^, ^86^] In the restricted interpretation, the word fuel is used exclusively for those cases in which the energy released from the chemical trigger is exploited by the system to carry out work. This implies that fuel consumption must drive the system to a non‐equilibrium composition. Within the Classes that we have identified in this Review, only Class 4 satisfies this criterion. In a much broader interpretation, though, the term fuel is used for any chemical stimulus that induces a transient change in the system as a result of conversion into waste, irrespective of whether this allows the system to perform work. Aware of this difference, throughout this Review we adhere to this broad interpretation because the usefulness of chemically fueled systems is not just limited to those systems that can exploit chemical energy to perform work (see Classes 2 and 3).

Before analyzing the system's response to the addition of a fuel, it is important to distinguish between situations in which the fuel is added batchwise and those in which the fuel concentration is kept constant. This represents the difference between a closed system and an open system in which fuel and waste are continuously exchanged with the environment. The energy diagram in Figure 5 b serves to visualize the composition of the system under stationary conditions, that is, in an open system. The green arrows indicate the processes that convert fuel into waste. Both start from the M and M_2_ (+F) energy levels, because fuel‐to‐waste conversion in Class 2 occurs after dissociation of F from M* and M*_2_. The red spheres indicate the relative population of each state and correspond to arbitrary values for *K*_1_–*K*_4_ used in previously reported simulations.^[62]^ For reasons of clarity, the populations of the M and M_2_ states are indicated just on the waste level (identical shaded spheres are inserted on the fuel level). Under stationary conditions, that is, at constant fuel and waste concentrations, the distribution is completely dictated by thermodynamics and M*_2_ is the most populated species. Importantly, because the self‐assembly and fuel‐to‐waste conversion processes are independent, energy consumption cannot affect the ratio between M and M_2_, which always corresponds to the ratio at thermodynamic equilibrium.

It is nonetheless possible to observe the high‐energy assembly M_2_, but only if fuel is added batchwise to the system and if assembly M_2_ has a high kinetic stability. Under these circumstances it is possible that assembly M_2_ temporarily persists in the system after the dissociation and destruction of F. The (spontaneous) switching of the environmental conditions (no fuel–fuel–no fuel) leads to a temporary alteration of the energy landscape and this can lead to the population of a high‐energy state. This mechanism of energy transfer—referred to as an energy ratchet—has been frequently applied to populate high‐energy states of molecular pumps.[^32^, ^49^, ^52^, ^87^] However, under stationary conditions this mechanism cannot be effective, as it intrinsically relies on the switching between different conditions. Indeed, we will show later that under stationary conditions M_2_ can be populated by an entirely different mechanism (Class 4).

Although the systems’ composition is determined by the same set of thermodynamic equilibria that govern Class 1, the transient change in the energy landscape upon the addition of a batch of fuel introduces the possibility of gaining temporal control over the composition. The extent at which the M*_2_ state is populated and the duration of this populated state can be controlled by the amount of fuel that is added; systems that operate according to this scheme are exploited for the activation of functions associated with the M*_2_ state with temporal and quantitative control.

### 3.2 Biology—Signal Transduction

An essential feature of a cell is the possibility to communicate with the extracellular environment by receiving and transmitting signals.[^88^, ^89^] Without communication, cells would not be able to organize themselves into structures of higher complexity.^[90]^ Communication relies on the activation of signal transduction pathways by messenger molecules that selectively bind to extracellular receptor sites of transmembrane proteins embedded in the cell membrane. Generally, the binding event leads to conformational changes or dimerization causing the activation of the intracellular domain for catalysis or the binding of intracellular components. Membrane receptors can be divided into three major groups based on the mechanism of signal transduction: G protein‐coupled receptors, ion channel receptors, and enzyme‐linked receptors. A single cell can have hundreds of different cell membrane receptors, which underlines the signaling power of a cell and the importance of communication.

**Figure 6:**
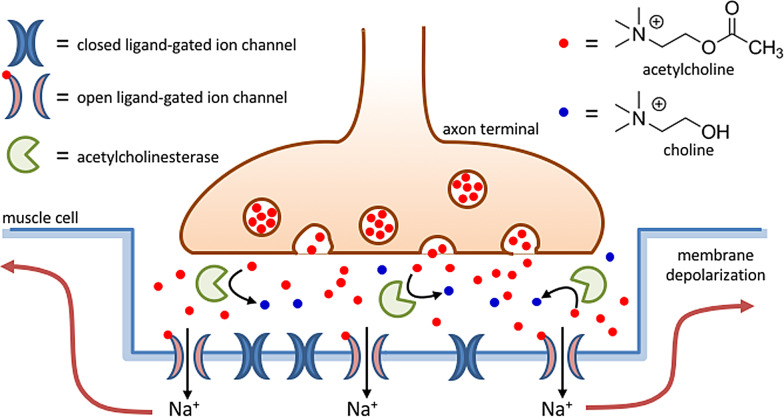
Schematic representation of a synaptic cleft at a neuromascular junction. Acetylcholine is released from vesicles in the axon terminus and causes opening of ligand‐gated ion channels in the muscle cell. At the same time, acetylcholine is rapidly cleared from the synaptic cleft by the enzyme acetylcholinesterase.

When studying signal transduction, the focus is generally on the signaling cascade that is triggered by the initial binding event. However, of equal importance is signal termination, as communication pathways are nearly always transient in nature. Signal termination requires elimination of the trigger, which can occur spontaneously through diffusion, but can also be an active process mediated by enzymes. Acetylcholine is a neurotransmitter that is released by nerve cells to send signals to other cells, such as other neurons and muscle cells. After release from the nerve cell, acetylcholine binds to receptor proteins embedded in the membrane of the receiving cell. Acetylcholine receptors can be divided into two major classes, nicotinic acetylcholine receptors (nAChR)^[91]^ and muscarinic acetylcholine receptors (mAChR),^[92]^ both of which respond to acetylcholine, but with different response mechanisms. Here, we focus in detail on nAChR, as it provides a fascinating example of how nature uses a chemical fuel—acetylcholine—under dissipative conditions to transiently activate function (Figure 6). At the neuromuscular junction, nAChR plays a key role in the transfer of signals from the nervous system to skeletal muscle cells. nAChR is a 290 kDa transmembrane protein made up of five subunits that are symmetrically arranged around a central pore.^[93]^ Binding of acetylcholine leads to a conformational change, which opens a channel that permits the permeation of Na^+^ and K^+^ ions through the membrane at a staggering rate of around 15 000–30 000 ions per millisecond.^[11]^ This induces a depolarization of the muscle membrane, which is the triggering event that leads to the release of Ca^2+^ ions stored intracellularly in the sarcoplasmic reticulum. The near‐instantaneous increase in the cytosolic Ca^2+^ concentration activates enzymes involved in muscle contraction (see also Section 5.1). Communication between the nervous system and skeletal muscles must be regulated at extremely short time intervals to allow an immediate reaction by the organism. This necessity is reflected by the rates of acetylcholine‐mediated activation of nAChR and its subsequent deactivation. Upon activation of the nervous system, acetylcholine is released in a burst‐wise fashion into the synaptic cleft from synaptic vesicles present in the nerve cell—each containing around 5000–10 000 molecules—causing an increase in the acetylcholine concentration in the synaptic cleft to around 0.3 mm.[^94^, ^95^] Acetylcholine binds nAChR with a relatively low dissociation constant (*K*_d_) of around 0.2 mm, which implies the binding sites are only partly occupied.^[96]^ Of importance is also the high dissociation rate constant of 5×10^4^ s^−1^, which implies acetylcholine occupies the binding site for very short times. Together, the low binding site occupancy and the short lifetime of the complex are essential to control ion‐channel opening and closure on the millisecond timescale and allows for a very fast deactivation pathway. Termination of the signal requires elimination of acetylcholine from the synaptic cleft. This role is carried out by the enzyme acetylcholinesterase, which cleaves acetylcholine into choline and acetate. Acetylcholinesterase is abundantly present in the synaptic cleft and cleaves acetylcholine at a rate of around 25 000–30 000 molecules per second, which approaches the limit set by substrate diffusion.^[97]^ This enzyme installs the dissipative conditions required to terminate the signal activation pathway by clearing the neurotransmitter from the synaptic cleft. In the context of this discussion, it is true that this example falls to some extent short in the sense that acetylcholine‐binding induces an intra‐ rather than intermolecular self‐assembly process in nAChR. However, we have selected this example because it demonstrates clearly how nature exploits dissipative conditions to temporally regulate chemical functions in an extraordinary way. Examples in which a chemical trigger transiently activates a signal transduction pathway by triggering changes in self‐assembly processes are abundant and involve, for example, G protein‐coupled receptors^[98]^ and integrin clustering on cell membranes.^[99]^

### 3.3 Chemistry—Transient Self‐Assembly of Nanoreactors

**Figure 7:**
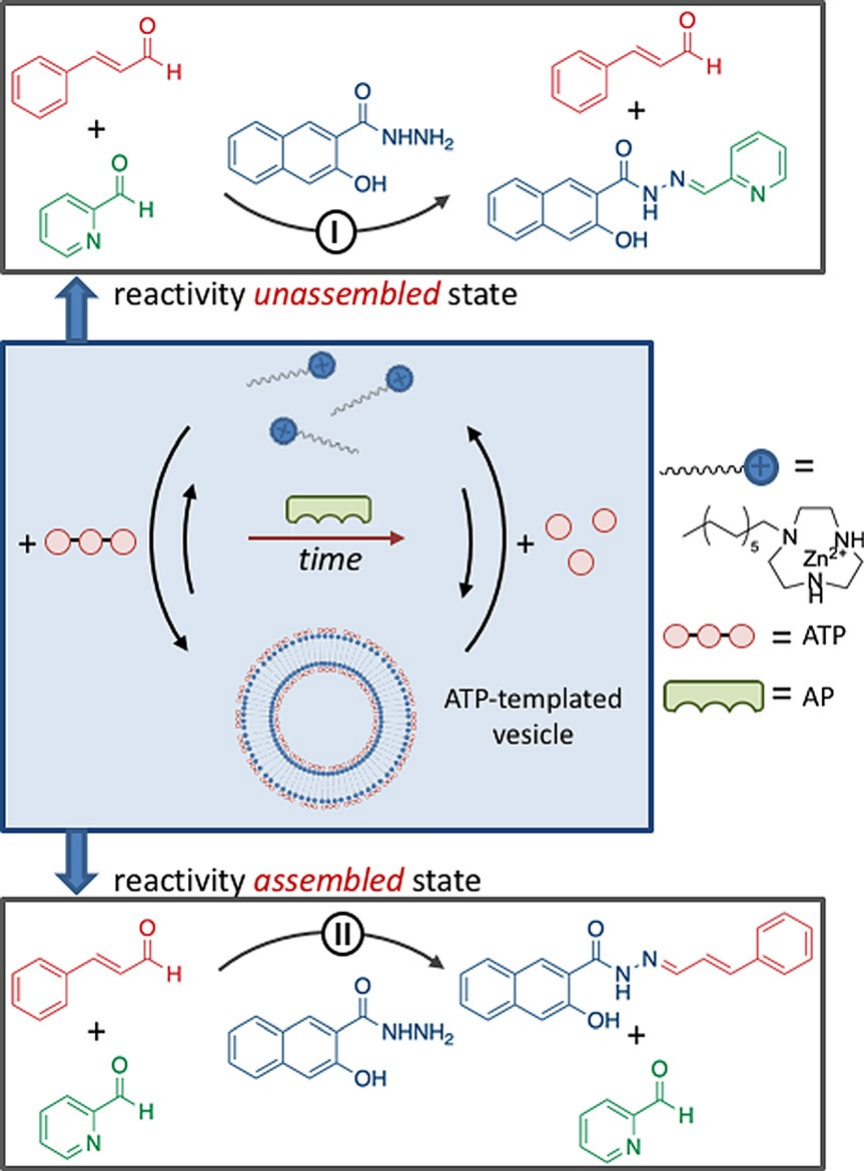
ATP‐templated self‐assembly of vesicles under dissipative conditions generated by the presence of the enzyme alkaline phosphatase. The assembly state affects the chemical reactivity in the system: reaction **I** dominates in the unassembled state, whereas reaction **II** dominates in the assembled state. Transient inversion of reaction selectivity is observed upon the addition of ATP.

In a chemical context, templated self‐assembly under dissipative conditions has emerged as a tool to control the lifetime of self‐assembled structures and, importantly, the functional properties exerted by templated assembly.[^58^, ^100^, ^101^, ^102^, ^103^, ^104^, ^105^] This requires that initial conditions are chosen such that the building blocks reside in the unassembled state (M) in the absence of fuel and that the waste molecules have no templating ability. Our group has illustrated how multivalency can be used to design a system that meets these criteria.^[106]^ Previously, we demonstrated that the interaction of adenine nucleotides (A*X*P, with *X*=M, D, or T) with the monolayer surface of gold nanoparticles covered with a monolayer of alkylthiols terminating with a 1,4,7‐triazacyclononane⋅Zn^2+^ (TACN⋅Zn^2+^) complex strongly increased as the number of phosphate groups present in the nucleotide increased.[^107^, ^108^] The multivalent effect originates from the establishment of multipoint interactions between the phosphate groups and Zn^2+^ metal ions embedded in the monolayer and the substitution of multiple—smaller—counteranions surrounding the monolayer with a single multivalent one. The observation of a strong multivalent effect in the nanoparticle system led us to explore its use for the templated self‐assembly of vesicles under dissipative conditions (Figure 7).^[109]^ The addition of ATP to a solution of C_16_TACN⋅Zn^2+^—in which the TACN⋅Zn^2+^ complex is attached to a saturated C_16_ chain—at a concentration below the critical micellar concentration resulted in the formation of ATP‐templated vesicles. However, the solution also contained potato apyrase, an enzyme that hydrolyzes ATP into AMP and two phosphate ions. As a consequence of the conversion of ATP into waste products, which are unable to stabilize the vesicles, the latter gradually dissociated in time. A new cycle of transient vesicle formation could be triggered by the addition of a new batch of ATP. In this system, the lifetime of the assemblies can be regulated by varying the concentration of ATP or the concentration of the enzyme.

The transient formation of ATP‐templated vesicles offers a new possibility to control chemical reactivity in the time domain.[^109^, ^110^, ^111^] Vesicles are characterized by an apolar bilayer, which can be used to trap apolar reactants that are poorly soluble in the aqueous medium.[^112^, ^113^] Uptake into a confined space leads to an increased local concentration, which favorably affects the reaction rates. The limited lifetime of the vesicles under dissipative conditions implies that a limited timeframe is available for observing such a rate acceleration. Transient upregulation of a chemical reaction was illustrated by using a system in which two hydrazone‐forming reactions **I** and **II** can take place contemporarily (Figure 7).^[110]^ The rate of reaction **I** is not affected by the presence of vesicles, whereas reaction **II**—which involves the apolar cinnamaldehyde—is characterized by a slow background reaction in water and a strong rate acceleration when vesicles are present. Reaction **I** dominates in a solution containing just the amphiphile, but the addition of ATP results in strong upregulation of reaction **II** and the corresponding hydrazone becomes the major product. However, reaction **II** gradually slows down as ATP is hydrolyzed by the enzyme and the product of reaction **I** becomes again the major species in the system. Inversion of the reaction selectivity can be retriggered by adding a new batch of ATP.

Templated assembly under dissipative conditions introduces the possibility to use time as a design criterion for developing complex systems. Although the distribution of species is still controlled by thermodynamics, the system responds dynamically to a gradual change in the fuel concentration. It must be noted, though, that this possibility requires the fuel to be added batchwise. In a hypothetical open system, the constant fuel concentration would ensure a permanent presence of the templated assembly and no difference from templated self‐assembly at equilibrium composition would be observed.

## 4 From Equilibrium to Non‐equilibrium Self‐Assembly

In our model self‐assembly process, the formation of M_2_ from M is energetically uphill. Therefore, the population of the M_2_ state—and the maintenance of that populated state—requires continuous consumption of energy. Analysis of Class 2 illustrates that in this case, it is not possible to exploit the chemical energy released during fuel‐to‐waste conversion to shift the equilibrium from M to M_2_ when the system is governed by a thermodynamic cycle. The reason for this is that self‐assembly and fuel‐to‐waste conversion in Class 2 are fully independent processes, which implies that energy transfer between these processes cannot take place. It follows that coupling of the self‐assembly and fuel consumption processes is required if the energy stored in chemical fuels is to be used to shift the equilibrium between M and M_2_. Classes 3 and 4 describe systems in which such a coupling is present. However, as we will discuss next, the coupling between self‐assembly and fuel consumption processes by itself is not sufficient to install a non‐equilibrium composition, which depends on the presence (Class 4) or not (Class 3) of kinetic asymmetry in the energy consumption pathway. As a result, fuel consumption changes the equilibrium composition between M and M_2_ in Class 4 (driven self‐assembly), but not in Class 3 (dissipative self‐assembly). It is noted that in our previous contribution, Classes 3 and 4 were referred to as symmetric and asymmetric dissipative self‐assembly, respectively.^[62]^

**Figure 8:**
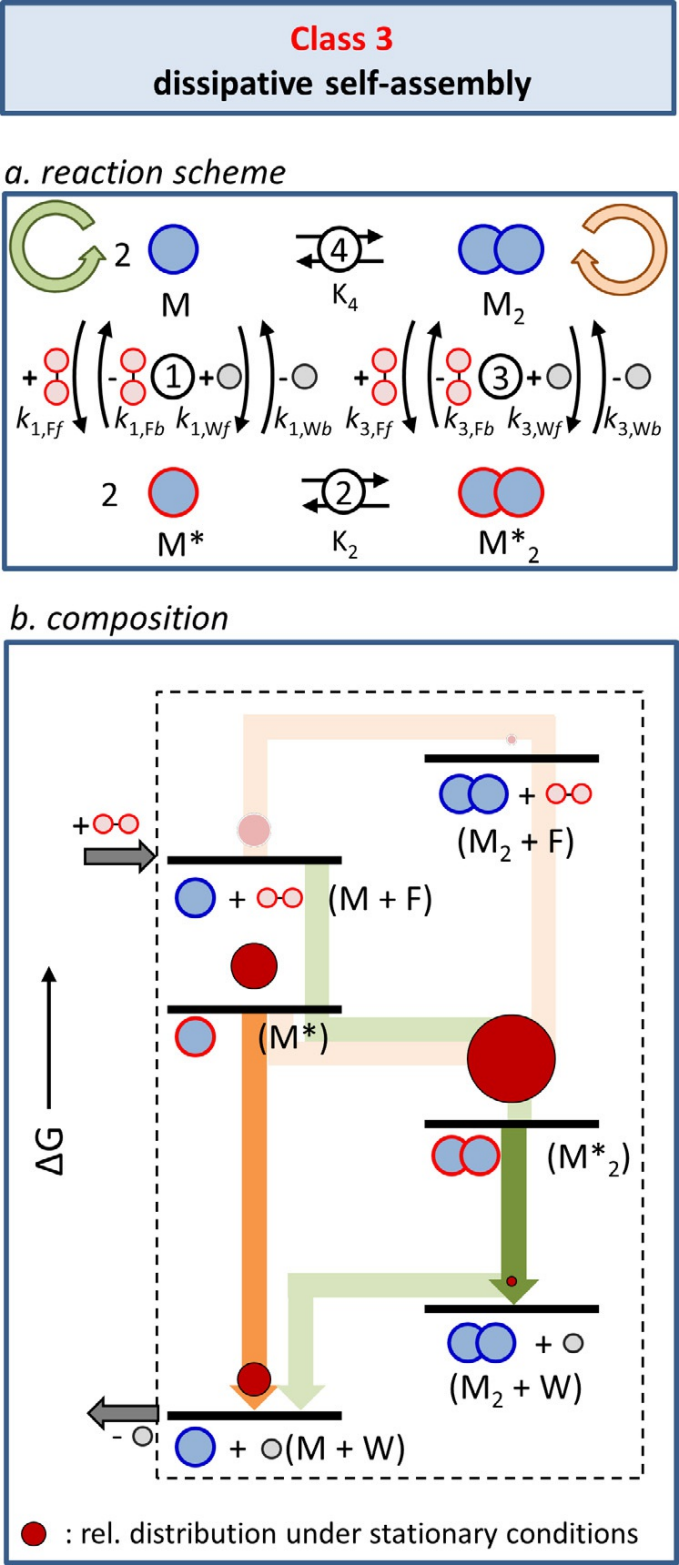
Class 3: Dissipative self‐assembly. a) Class 3 is characterized by the presence of two chemical connectivities between the non‐activated and activated building blocks involving either fuel or waste. b) Energy diagram illustrating the composition of the system under stationary conditions, that is, at constant fuel and waste concentrations in an open system. The orange and green trajectories represent the clockwise (orange) and counterclockwise (green) directions of the cycle depicted in Figure 8 a. The dark colored arrows correspond to the processes during which waste molecules are generated. The size of the dark red circles indicates the relative populations of the respective states reported in previous simulations.^[62]^

The difference between Classes 2 and 3/4 is immediately clear from a comparison of the reaction schemes (Figure 5 a: Class 2, Figure 8 a: Class 3, Figure 11 a: Class 4). In Classes 3 and 4, an additional connection exists between M and M_2_ and their activated counterparts M* and M*_2_, respectively, which involve the waste. This new connection implies that the self‐assembly components play a role in the conversion of fuel into waste. This can occur in two ways depending on whether the fuel interacts in a covalent or noncovalent manner with the building blocks. In the first case, a chemical reaction between M and F leads to product M*, which self‐assembles to give M*_2_ in a thermodynamically driven process. A second, different reaction converts M* back into M and generating waste. Similar activation and deactivation pathways are present on the other side of the equilibrium involving the interconversion between M_2_ and M*_2_. Alternatively, in cases where the interaction between the fuel and the building blocks is noncovalent, M (and M_2_) play the role of catalysts for the fuel–waste conversion. Complex formation between M (or M_2_) and F leads to the activated complex M* with the capacity to catalyze the conversion of fuel into waste.

The presence of two chemically distinct pathways between M and M* (and M_2_ and M*_2_) is essential for overcoming the problem of detailed balance, which prevents any non‐equilibrium composition of a thermodynamic cycle (Class 1) under stationary conditions. Indeed, because of the presence of two pathways in steps 1 and 3, the distribution of species in the system is now defined by Equation 2.

(2) $${\left(\frac{k_{1,\;Ff}+k_{1,\;Wf}}{k_{1,\;Fb}+k_{1,\;Wb}}\right)}^{2}K_{2}{\left(\frac{k_{3,\;Ff}+k_{3,\;Wf}}{k_{3,\;Fb}+k_{3,\;Wb}}\right)}^{-1}K_{4}^{-1}=K_{r}$$

Here, the equilibrium constants for steps 1 and 3 have been replaced by the kinetic constants for the forward (*f*) and backward (*b*) reactions involving fuel (F) and waste (W). It should be noted that some of the rate constants are apparent first order rate constants that include the concentrations of fuel and waste.^[62]^ The fundamental difference with the thermodynamic cycle is that the product of the terms on the left‐hand side of the equation does not have to be equal to 1, which is a direct consequence of the fact that the fuel‐to‐waste conversion releases energy into the system. This implies that—in contrast to the thermodynamic cycles of Class 1—detailed balance can be broken. Under stationary conditions, the rates of the forward and backward reactions in equilibria 4 and 2 may be different, thereby leading to currents, which are defined as the difference between the forward and backward rates. The presence of currents implies that more molecules follow the cycle in one direction than the other. We will see that the reason for this preferential directionality is that the energy barriers encountered in one direction are lower than the energy barriers encountered when following the cycle in the opposite direction. The product of the terms in Equation (2) can be defined as a ratcheting constant, *K*_r_, which can be interpreted as a directionality parameter that indicates whether a preference exists for the counterclockwise (*K*_r_ >1) or clockwise direction (*K*_r_ <1). For *K*_r_=1, no preferential directionality is present.

## 5 Class 3: Dissipative Self‐Assembly (*K*_r_=1)

### 5.1 Concept

The importance of the ratcheting constant emerges from simulations that we have reported previously.^[62]^ In a first set of simulations we analyzed the composition of the system in cases where the rate constants for the fuel–waste reactions 1 and 3 were imposed such that *K*_r_=1; in this case the system is said to display kinetic symmetry. The result of kinetic symmetry is that in a stationary state—that is, at constant fuel and waste concentrations in an open system—at any given time, the number of molecules that follow the clockwise pathway (orange) is equal to the number of molecules that follow the counterclockwise pathway (green; Figure 8 a). Both trajectories are also indicated in the corresponding energy diagram (Figure 8 b). Under these conditions, a situation is installed that is similar to the thermodynamic cycle of Class 1; no currents are present in the system. Indeed, simulations show that in the case of kinetic symmetry, the composition of the system is entirely dictated by the relative thermodynamic stabilities, and the assembled structure M*_2_—the thermodynamically most stable species—is the most populated species in the system under stationary conditions (Figure 8 b). Importantly, for *K*_r_=1, the rates at which M and M_2_ are consumed and reformed as a result of the activation (+F) and deactivation (‐W) pathways are such that the ratio between M and M_2_ always corresponds to the ratio at thermodynamic equilibrium. In other words, in a system that displays kinetic symmetry, energy is continuously being dissipated, but without affecting the composition of the system, which remains fully dictated by the relative thermodynamic stabilities.

### 5.2 Biology—Smooth Muscle Contraction

As an illustration of a biological dissipative self‐assembly process with kinetic symmetry, we have chosen an example of the most common way chosen by nature to regulate chemical processes—the reversible phosphorylation of proteins.^[114]^ Protein phosphorylation is the covalent post‐translational modification of proteins in which an amino acid residue (Ser, Tyr, or Thr) is phosphorylated through a kinase‐catalyzed transfer of the γ‐phosphate group of ATP. A second class of enzymes, phosphatases, reverses this modification by catalyzing the opposite dephosphorylation reaction. Protein phosphorylation plays a role in nearly all cellular functions, ranging from enzyme activation, signal transduction, subcellular localization, to apoptosis.^[115]^ The widespread use of this post‐translational modification as a regulatory tool emerges from the fact that the human genome encodes roughly 500 kinases and 200 phosphatases, which have been estimated to act on over 13 000 human proteins.^[116]^ The use of reversible protein phosphorylation as a switch is attractive for several reasons. Post‐translational modification implies that activation and deactivation does not require the synthesis or degradation of the protein. The phosphate group can be rapidly introduced and removed. The selectivity of kinases in terms of substrate recognition implies that, in principle, a single energy currency—ATP—can be used to drive the entire biological machinery. Phosphorylation of key amino acids leads to the introduction of a highly polar phosphate group with negative charge, which can induce structural reorganization of the protein or affect the interaction with other proteins. Reversible phosphorylation thus succinctly illustrates how nature uses building block activation by chemical fuels to exert temporal control over chemical functions. The concentration of the activated building blocks is determined by the relative rates of the chemically distinct forward and backward reactions which are regulated by kinases and phosphatases, respectively.

In line with the focus of this work on structural organization, we have chosen to discuss in detail an example in which the phosphorylation state of the protein affects its self‐assembly properties. On the molecular level, muscle contraction is caused by the ATP‐driven sliding of tiny filaments composed of the proteins actin and myosin II.^[11]^ In cardiac and skeletal muscle cells, these filaments are arranged in well‐defined contractile units, called sarcomeres, which line up and are bundled into myofibrils, thereby giving a characteristic striated appearance when imaged under a microscope.^[117]^ In these cells, contraction is activated by a sudden rise in the cytosolic Ca^2+^ concentration triggered by a signal from the nervous system (Section 3.2). The increase in the Ca^2+^ concentration is transient because Ca^2+^ is rapidly pumped outside of the cells; within around 30 ms the cytosolic Ca^2+^ concentration is restored and the myofibrils relax. The structural organization of actin and myosin and the Ca^2+^‐regulatory mechanism in these cells have evolved to permit the fast generation of force. In contrast, smooth muscle tissue is responsible for the slow and sustained contraction of, amongst others, the intestines, artery walls, and the uterus.^[118]^ This different function is reflected by the lower structural order of actin and myosin—the name smooth derives indeed from the fact that these cells lack the striated appearance of cardiac and skeletal muscle cells—and possess a different (slower) activation mechanism that relies on the phosphorylation of myosin. A similar mechanism also regulates myosin activity in non‐muscle cells and provides control over the actin cytoskeleton present in nearly all eukaryotic cells.

**Figure 9:**
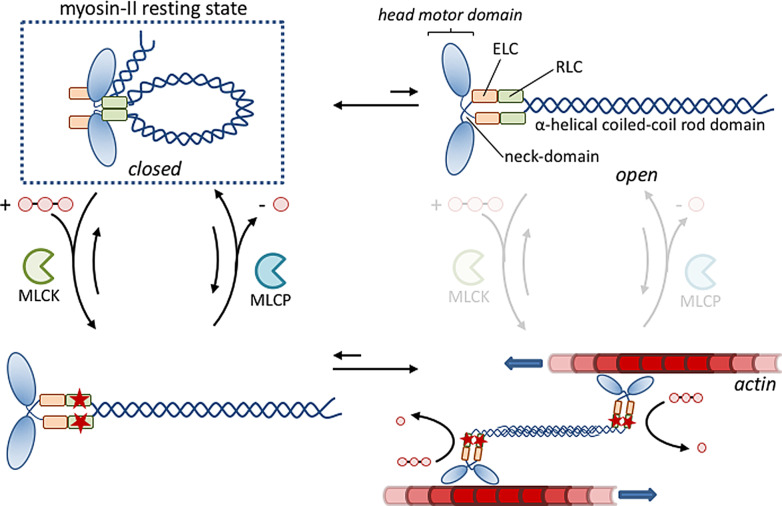
Schematic representation of the enzyme‐regulated activation and deactivation of myosin II. Figure inspired by Ref. [121].

Myosin II in smooth muscle cells and non‐muscle cells are structurally very similar (Figure 9).^[119]^ Myosin II is composed of three pairs of peptides—two heavy chains (230 kDa), two regulatory light chains (RLCs, 20 kDa), and two essential light chains (ELCs, 17 kDa)—and several domains are notable in the myosin II structure.^[120]^ Two globular head domains are involved in binding to actin and each of them contains a catalytic site for hydrolysis by ATP, which provides the energy for myosin II movement along the actin tracks. ATP binding and hydrolysis reorganizes the neck domain, which generates motion in a similar way as kinesin, which will be discussed later in detail (Section 8.2). Each neck domain also binds the RLC and ELC light chains. Finally, a long α‐helical coiled‐coil rod domain is present which ensures dimerization of the two heavy chains.

Conversion of the energy stored in ATP‐activated myosin into movement along the actin track during contraction requires crosslinking between different actin tracks. In cardiac and skeletal muscles, the permanent organization of myosin and actin filaments in sarcomeres ensures that these muscle cells are always ready for contraction. In contrast, this permanent predisposition of myosin within myofilaments is absent in smooth muscle and non‐muscle cells. Indeed, in these cells, myosin adopts a compact, folded conformation through a head‐to‐tail interaction.^[121]^ In this resting state, myosin has a low affinity for actin and does not self‐assemble into filaments. Myosin II activation requires phosphorylation of residue Ser19 on the regulatory light chain (RLC) by myosin light‐chain kinase (MLCK). In vitro experiments have shown that phosphorylation opens up the myosin II structure, which leads to actin‐binding by the head domains and, importantly, self‐assembly of myosin II into filaments through association of rod domains.^[122]^ MLCK activity requires binding of the Ca^2+^‐calmodulin complex, which implies that, just as in cardiac and skeletal muscles, contraction is regulated by Ca^2+^ ions. However, the kinase activation pathway is much slower and maximum contraction can require almost a second compared to a few milliseconds for striated muscle cells.^[11]^ A second major difference with the striated muscle cells occurs in the relaxation phase. Whereas striated muscle relaxes within milliseconds after contraction because of the rapid reduction in the cytosolic Ca^2+^ concentration, smooth muscle remains in the contracted form as long as myosin II remains phosphorylated. Dephosphorylation catalyzed by myosin light‐chain phosphatase (MLCP) makes myosin return to the folded conformation, and dissociation of the filaments takes place.^[123]^ Although much slower compared to the activation and deactivation of striated muscle, this type of regulation is better suited to serve the purpose of smooth muscle, which is to maintain force for a prolonged period of time with minimal energy consumption.

The operating mechanism of myosin II makes sense within the framework of Class 3. Protein (de‐)phosphorylation controls the ability of myosin II to self‐assembly and, consequently, exert its biological function. However, the self‐assembly of phosphorylated myosin II (M* in Figure 8 a) itself is a thermodynamically controlled process. This permits the structure (M*_2_) to be maintained without additional energy consumption. For the biological function of myosin II, the hypothetical use of a high‐energy assembly M_2_ would not add any value, but rather would imply an unnecessary expense of energy.

### 5.3 Chemistry—Transient Self‐Assembly of Hydrogels

**Figure 10:**
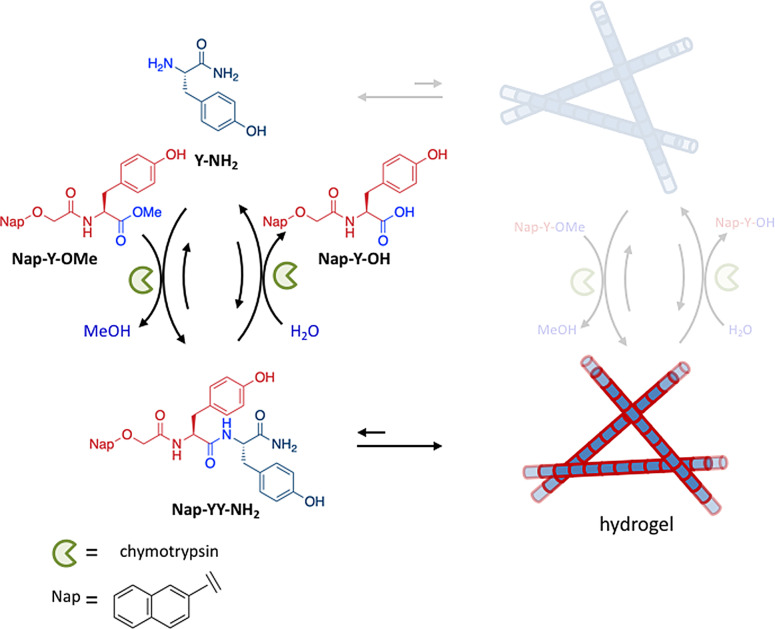
Transient self‐assembly of a hydrogel caused by the enzyme‐mediated activation and deactivation of C‐terminal‐amidated l‐tyrosine (Y‐NH_2_).

In recent years, the development of synthetic chemically fueled self‐assembly processes that mimic the phosphorylation‐dephosphorylation scheme of biological fuel‐activated processes has truly taken flight.[^124^, ^125^, ^126^, ^127^, ^128^, ^129^, ^130^, ^131^, ^132^, ^133^, ^134^, ^135^, ^136^] Many systems have now been described that rely on the fuel‐mediated activation of building blocks for self‐assembly under conditions where a concurrent and chemically distinct pathway causes deactivation. It was quickly realized that this provides a way to form nanostructures and materials with a controlled lifetime, which is not possible for thermodynamically controlled self‐assembly processes. In the vast majority of cases, however, the attention has been primarily focused on the self‐assembly process and the properties of the thermodynamically stable assembly composed of activated building blocks, which corresponds to assembly M^*^_2_ in our scheme. The presence of high‐energy assemblies composed of non‐activated building blocks, that is, M_2_, has so far not been specifically searched for, and detailed kinetic studies that would prove the installation of kinetic asymmetry have not been reported. In the absence of this information it is, therefore, not possible to unequivocally assign the reported systems to Class 1 or 2. Here, we discuss a system reported by Ulijn and co‐workers for which no particular properties have been reported that would indicate the formation of a high‐energy assembly (Figure 10).^[124]^ In addition, the fact that activation and deactivation of the building blocks occurs in the active site of enzymes combined with the fact that bond making and breaking involves an internal bond of the building block, make it more likely that the conversion of fuel into waste involves the free building blocks rather than the assembled ones.

The reported system exploits the ability of proteases to catalyze the formation (rather than hydrolysis) of peptide bonds under specific experimental conditions. For example, chymotrypsin efficiently catalyzes the formation of a peptide bond when an ester precursor is used, rather than a carboxylic acid. This property was exploited for the transient formation of a hydrogel from dipeptide gelators. C‐Terminal‐amidated l‐tyrosine (Y‐NH_2_) was used as a building block and converted into a dipeptide gelator upon reaction with the methyl ester of l‐tyrosine, functionalized at the N‐terminus with an apolar naphthoxyacetyl group (Nap‐Y‐OMe). The latter species can be described as a chemical fuel, because this compound drives the process and eventually ends up as a carboxylic acid waste molecule. The chymotrypsin‐catalyzed reaction between the building block and the fuel leads to the rapid formation—within 2–3 minutes—of the dipeptide Nap‐YY‐NH_2_. This dipeptide has a strong tendency to assemble into nanofibers through a combination of π‐π stacking interactions between the naphthyl groups and hydrogen bonding between the amide bonds. The self‐assembly of fibers is evidenced on the macroscopic level by hydrogel formation. However, over time, chymotrypsin slowly hydrolyzes the amide bond between the tyrosine residues in Nap‐YY‐NH_2_ to yield the original building block Y‐NH_2_ and the waste molecule Nap‐Y‐OH. This reaction is actually an equilibrium, but the composition at equilibrium lies so far to the product side that the final concentration of Nap‐YY‐NH_2_ is negligible. It was shown that transient hydrogels were obtained in cases where the gelator Nap‐YY‐NH_2_ could be produced and maintained above the critical gelator concentration for a certain time interval before the final state was eventually reached. Importantly, the lifetime of the gel could be controlled by regulating the kinetics of the chemical reactions (pH, enzyme concentration), which is an important general feature of systems that display dissipative self‐assembly.

Many conceptually related examples have followed this early report and have convincingly demonstrated that the properties of materials, amongst others, can indeed be controlled using a simple set of orthogonal chemical reactions.[^124^, ^125^, ^126^, ^127^, ^128^, ^129^, ^130^, ^131^, ^132^, ^133^, ^134^, ^135^, ^136^] This bears a strong resemblance to the activation of biological functions through transient chemical modification of proteins. Chemical fuel is consumed, but the released energy is not transferred to the self‐assembly process in the sense that it does not lead to the population of a high‐energy assembled state. Indeed, from a conceptual point of view, no new properties appear compared to thermodynamically controlled templated self‐assembly under dissipative conditions.

## 6 Class 4: Driven Self‐Assembly (*K*_r_≠1)

### 6.1 Concept

**Figure 11:**
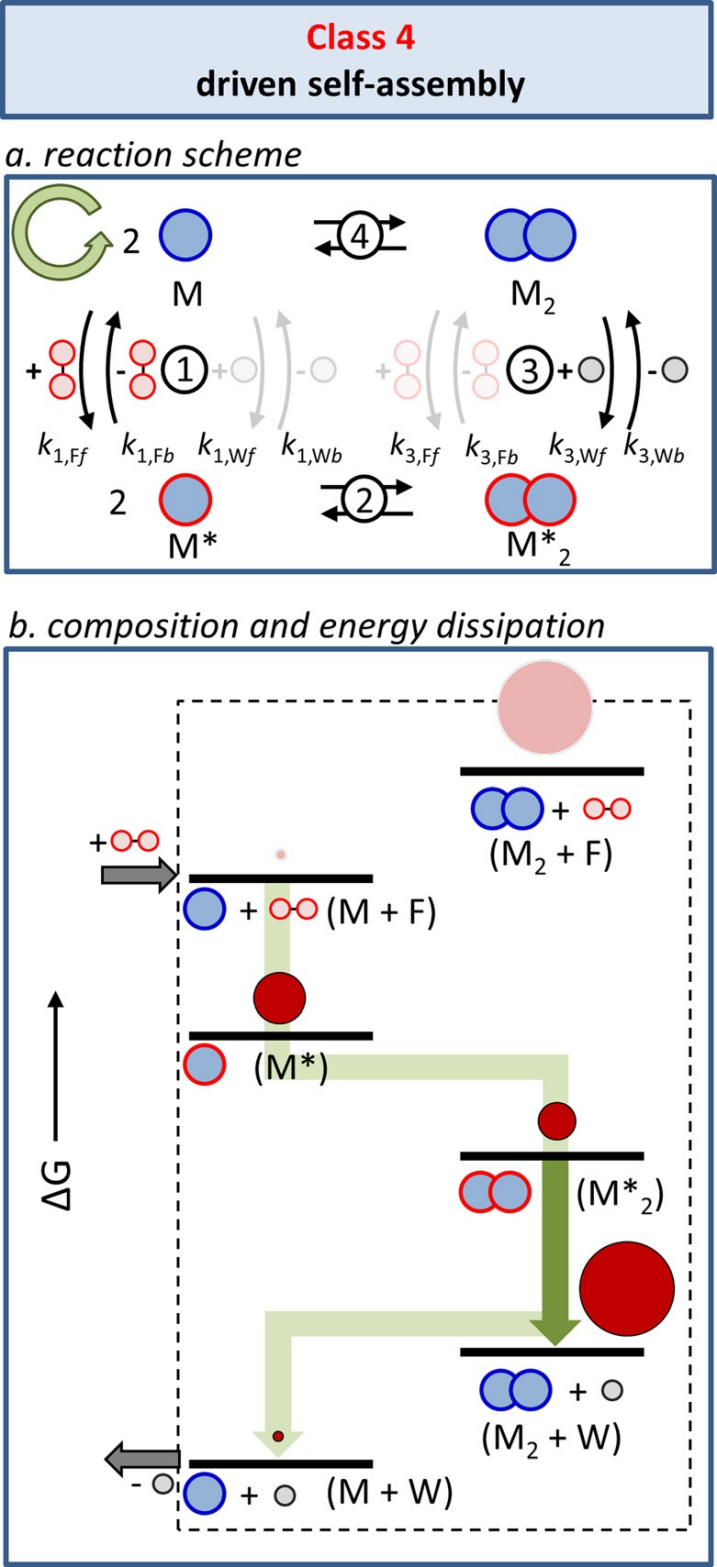
Class 4: Driven self‐assembly. a) Kinetic asymmetry arises from the presence of different energy barriers for the fuel and waste reactions with respect to the unassembled (M) and assembled (M_2_) states. b) Energy diagram illustrating how kinetic asymmetry creates the conditions required for the permanent population of the high‐energy assembly M_2_ under stationary conditions. The green trajectory represents the counterclockwise (green) direction of the cycle depicted in Figure 11 a. The dark green arrow corresponds to the process during which waste molecules are generated. The size of the dark red circles indicate the relative population of the respective state reported in previous simulations.^[62]^

A radically different situation is obtained in cases where kinetic asymmetry is present in the system (*K*_r_≠1; Figure 11). This emerged from a second simulation that we reported previously,^[62]^ in which the rate constants for the fuel‐ and waste‐mediated reactions 1 and 3 were imposed in such a way that *K*_r_>1. This value for the ratcheting constant implies that, under stationary conditions, more molecules follow the cycle in the counterclockwise direction (green arrow) compared to the clockwise direction (orange arrow). Such kinetic asymmetry was achieved by lowering the energy barriers for equilibria 1F and 3W (simulated by increasing *k*_1,F*f*_ *, k*_1,F*b*_, *k*_3,W*f*_, and *k*_3,W*b*_) and by increasing the energy barriers for equilibria 1W and 3F (simulated by decreasing *k*_1,W*f*,_ *k*_1,W*b*_, *k*_3,F*f*_, and *k*_3,F*b*_). In practice, this implies that the fuel reacts faster with M than M_2_ and that waste is more rapidly produced by M*_2_ than M*. Importantly, this creates a preferential pathway (green) that includes the formation of the high‐energy assembly M_2_ from M*_2_ (Figure 11 b) as a key passage in the energy dissipating process. Indeed, simulations show that, as a result of kinetic asymmetry, the high‐energy assembly M_2_ becomes the most populated species in the system under stationary conditions. It should be noted that this requires fuel‐to‐waste conversion to occur more rapidly than the equilibration in steps 2 and 4 (for a discussion on the importance of kinetic stabiity of assembly M_2_ see also Section 9). The observation that equilibrium 4 between M and M_2_ has shifted towards the energetically uphill assembly of M_2_ unequivocally demonstrates that the energy released from fuel‐to‐waste conversion is used to sustain the stationary non‐equilibrium composition of the system. The same insight emerges from an analysis of the kinetically preferred pathway in the system [Eqs (3)–5]:

(3) $$2\;M+2\;F\vec{\leftarrow }2\;M{}^{*}$$

(4) $$2\;M{}^{*}\vec{\leftarrow }M{}^{*}{}_{2}$$

(5) $$M{}^{*}{}_{2}\vec{\leftarrow }M{}_{2}+2\;W$$

This set of equilibria provides, as sum, the overall equilibrium is described by Equation 6:

(6) $$2\;M+2\;F\vec{\leftarrow }M{}_{2}+2\;W$$

This equilibrium illustrates that the self‐assembly of M to form M_2_ is now coupled to fuel consumption, thus permitting a transfer of energy between the two processes. Kinetic asymmetry in the system creates a situation in which a chemical fuel drives the self‐assembly of M into M_2_. In this case, the identification of the high‐energy molecule as a “fuel” that drives the system to a non‐equilibrium state leaves no room for ambiguity.^[41]^

### 6.2 Biology—Microtubule Formation

Microtubules are noncovalent protein polymers that play an essential role in intracellular organization. Microtubule filaments give structure and shape to eukaryotic cells, serve as tracks for intracellular transport, and are involved in chromosome segregation during cell division. Microtubule networks are highly dynamic structures which are continuously being remodeled through stochastic length fluctuations at the ends of the individual microtubules, a property referred to as dynamic instability.[^137^, ^138^] Dynamic instability manifests itself through periods of persistent microtubule growth which are occasionally interrupted by moments of rapid shrinkage (called “catastrophe”), followed by a switch back to polymer growth (called “rescue”). We will illustrate here that dynamic instability is a property that originates from kinetic asymmetry in the energy consumption pathway of this chemically fueled self‐assembly process.

**Figure 12:**
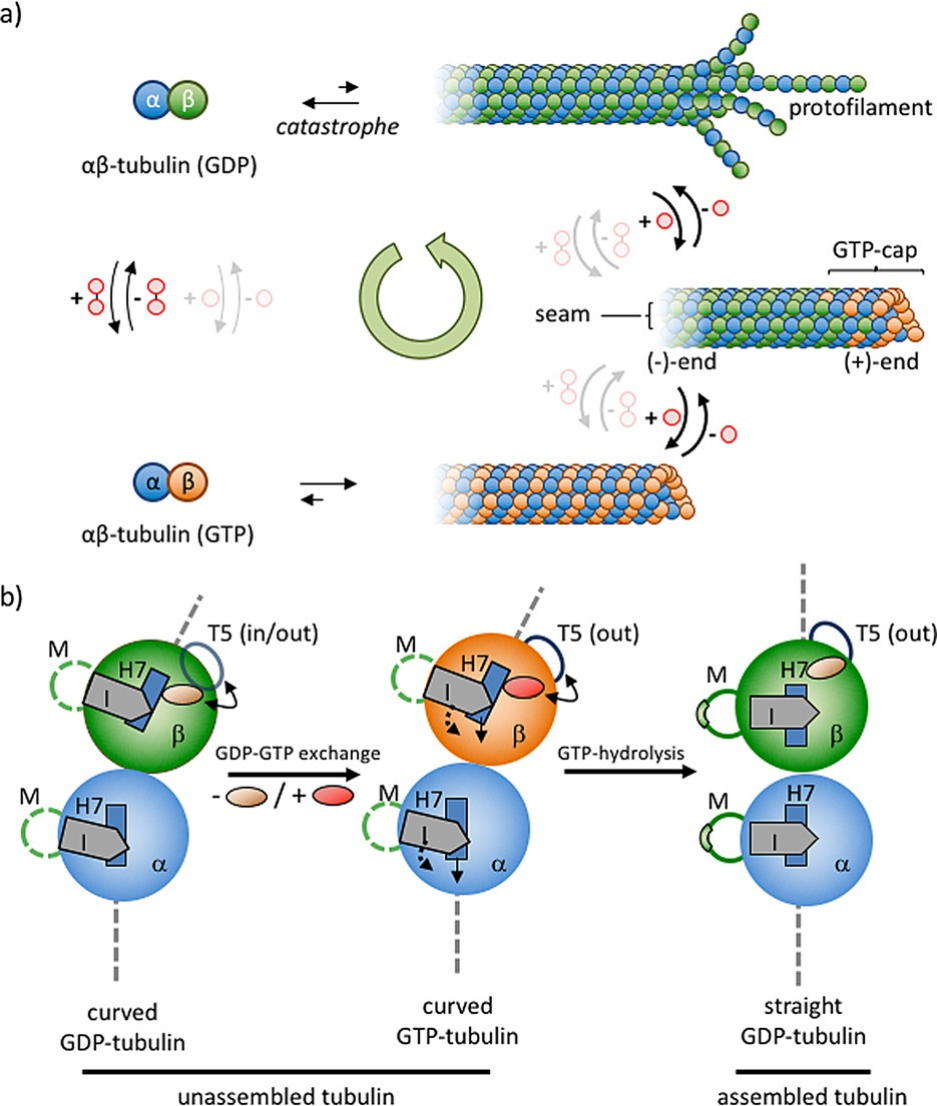
a) GTP‐fueled self‐assembly of microtubules. b) Structural changes in the αβ‐tubulin dimer as a function of the occupancy of the E‐site with GDP or GTP. Conversion of GTP into GDP in assembled tubulin leads to strain in the microtubule lattice. Figure inspired by Ref. [144].

Microtubules are stiff, nanotubular structures with an outer diameter of around 25 nm (Figure 12).[^19^, ^139^] The subunit of microtubules is a heterodimer of α‐ and β‐tubulin proteins—each with a mass of around 50 kDa—which associate in a head‐to‐tail manner to form linear structures called protofilaments. Typically, 13 such protofilaments engage in lateral interactions to form a microtubule. In the microtubule lattice, lateral interactions between protofilaments are α to α and β to β, except at the seam, where α‐tubulin binds β‐tubulin. As a result of the longitudinal arrangement of αβ‐tubulins in protofilaments, microtubules have one end (−) where the α‐subunits are exposed, and one end (+) where the β‐subunits are exposed. Frequently, the (−)‐end of microtubules is anchored to microtubule organizing centers, such as the centrosome. Dynamic instability occurs predominantly at the (+)‐end of microtubules.

Microtubule assembly and disassembly is regulated by guanosine triphosphate (GTP; Figure 12 a). Both α‐ and β‐tubulin have a binding site for GTP, but these sites have very different properties and functions.^[140]^ The binding site in α‐tubulin (N‐site) is buried within the tubulin dimer at the interface between α‐ and β‐tubulin. GTP bound to the N‐site plays a structural role and is non‐exchangeable and non‐hydrolysable. On the other hand, the binding site in β‐tubulin (E‐site) is exposed on the surface of free tubulin and on the terminal tubulin subunits located at the (+)‐end of microtubules. The properties of this binding site change dramatically upon the incorporation of free tubulin onto the microtubule. With free tubulin, nucleotide exchange at the E‐site is possible and the bound GTP is chemically stable. However, upon inclusion in the microtubule, nucleotides bound to the E‐site become non‐exchangeable and GTP is rapidly converted into GDP+P_i_.[^141^, ^142^] The difference in the properties of the E‐site between free and polymerized tubulin constitutes the basis for kinetic asymmetry in this process and for that reason it is relevant to discuss what happens upon polymerization from a structural point of view.

Microtubule formation initiates with an exchange of GDP by GTP in free tubulin. GDP‐tubulin does not polymerize, but the exchange of GDP with GTP causes a conformational change in the T5 loop of β‐tubulin, which activates tubulin for polymerization (Figure 12 b).[^143^, ^144^] Polymerization of GTP‐tubulin is a thermodynamically favored process, which is evidenced by the fact that the use of non‐hydrolysable analogues of GTP results in the formation of thermodynamically stable microtubules that do not display dynamic instability.^[145]^ Two important processes occur when GTP‐activated tubulin attaches to the microtubule (+)‐end. Firstly, lateral and longitudinal interactions with the microtubule lattice cause a straightening of the tubulin, which in the free form is curved with a 12° kink at the dimer interface. Secondly, binding to the polymer activates the E‐site for the catalytic cleavage of GTP into GDP+P_i_, which occurs rapidly after association of GTP‐tubulin with the microtubule following first order kinetics . Consequently, microtubules are composed mostly of GDP‐tubulin, even though that unit by itself does not polymerize under equilibrium conditions. Importantly, the conversion of GTP into GDP compacts the tubulin structure, leading to an increase in lattice strain in the microtubule. The presence of lattice strain implies that these are high‐energy structures composed of “spring‐loaded” GDP‐tubulin. The reason why the structure does not collapse originates from the presence of a stabilizing GTP cap at the (+)‐end of the microtubule. Although the precise nature of the GTP cap is still under debate, there is consensus that the microtubule end must be regarded as a dynamic structure, where the amount of GTP‐tubulin depends on the availability of free GTP‐tubulin.^[146]^ A reduced influx lowers the amount of stabilizing GTP‐tubulin in the cap and increases the possibility that the entire microtubule will collapse (“catastrophe”).

Within the context of this Review, it is of relevance to recognize the elements that install kinetic asymmetry in the cycle that describes GTP‐fueled microtubule formation. The observation that GDP–GTP exchange can only occur in the E‐site of free tubulin implies that fuel activation involves only the building block and not the assembly. In addition, only the E‐site of assembled tubulin is catalytically active, which implies that energy dissipation leads to the formation of a high‐energy assembly composed of GDP‐tubulin. Consequently, a kinetic preference for counterclockwise directionality exists in the system, which shows that GTP–GDP conversion and microtubule self‐assembly are coupled processes. The structural analysis illustrates that the energy released from GTP is stored as lattice strain in the GDP‐rich assembly.

### 6.3 Chemistry—Self‐Assembled Fibers with Dynamic Instability

The installation of kinetic asymmetry has so far rarely been considered in the design of synthetic dissipative self‐assembly processes. As discussed above, the main focus has been on the formation of the self‐assembled structure, which in nearly all cases regards the thermodynamically stable self‐assembly corresponding to M*_2_ in our general scheme. In most cases, little attention has been paid to the potential presence of the high‐energy assembly M_2_, although, as we have seen in this section, it is the fuel‐driven formation of this species that leads to valuable properties, such as the dynamic instability of microtubules. However, although kinetic asymmetry has not been actively sought for, experimental observations strongly suggest that some systems behave in a similar manner as microtubules.

**Figure 13:**
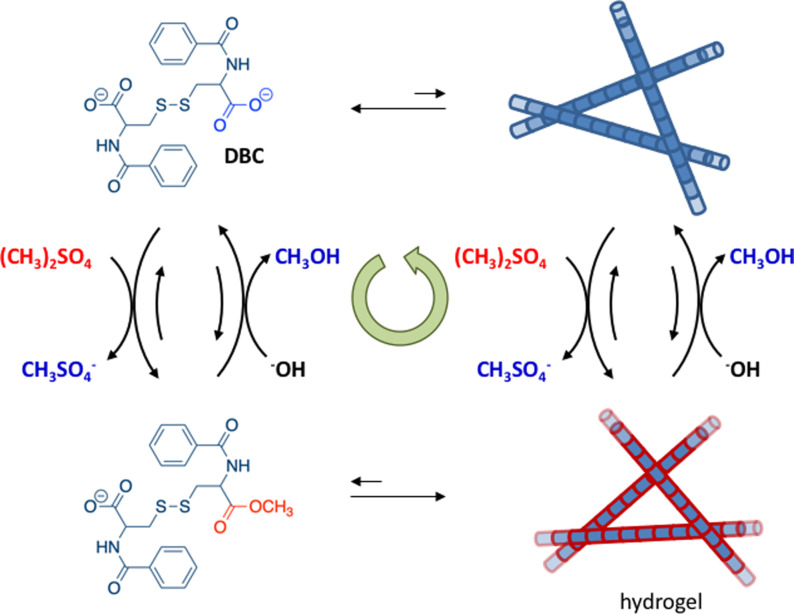
Chemically fueled driven self‐assembly of fibers that show features reminiscent of the catastrophic collapse of microtubules.

Van Esch and co‐workers reported on the chemically fueled self‐assembly of fibers which displayed properties that strongly resembled the dynamic instability of microtubules (Figure 13).[^147^, ^148^] This system relied on the activation of *N*,*N*′‐dibenzoyl‐l‐cysteine (DBC) for self‐assembly by means of an esterification reaction with dimethyl sulfate. DBC by itself did not assemble under the experimental conditions (basic pH) because of electrostatic repulsion between the carboxylate groups. The addition of dimethyl sulfate, which is a strong methylating agent, caused the formation of the monoester of DBC, which self‐assembled into bundles of fibers with micrometer dimensions, thereby leading to gelation of the aqueous solution. However, at the high experimental pH values, gradual hydrolysis of the methyl ester took place, which caused re‐formation of the starting compound DBC, and spontaneous dissolution of the gel was observed over time. Similar to the example discussed in the Section 5.3, it was observed that the lifetime of the gel could be regulated by tuning the rates of the forward (fuel concentration) and backward reactions (pH).

Two observations strongly suggested that the properties of the system were not solely governed by the thermodynamically stable fibers (M*_n_) composed of the monoester of DBC, but that also the unactivated building block DBC played a role in the self‐assembly process. Firstly, a delay was observed between changes in the concentration of the monoester and the rheological behavior of the gel in the decay phase. For example, at pH 11, a gel state was still observed after 10 hours, whereas the concentration of the monoester had already dropped to 0.6 mm after 5 hours. The second observation came from a confocal microscopy study, which provided insight into the behavior of single fibers during the dissipative cycle. The addition of dimethyl sulfate at pH 11 resulted in the rapid formation of fibers, which reached a maximum length in a time that corresponded with the time required for the gel to reach the maximum storage modulus. Next, the fibers entered a shrinking regime and decreased in length. However, rather than the expected gradual and simultaneous shrinking of all fibers, a stochastic abrupt collapse of the fibers was observed, reminiscent of the collapse of microtubules. The fibers shrunk only from their ends; a fracturing or homogeneous dissolution of the fibers was not observed. A time regime existed in which the growing and shrinking of fibers occurred simultaneously. Altogether, these observations are coherent with the formation of high‐energy fibers that are (partially) composed of the non‐activated building block DBC, obtained through the hydrolysis of the methyl esters of the building blocks in the fibers. The strong similarity with the dynamic instability of microtubules suggests that the fuel‐driven cycle is regulated by a similar kinetic scheme. A detailed kinetic analysis of the activation and deactivation reaction steps would serve to unequivocally demonstrate the presence of kinetic asymmetry in this system.

## 7 Information Ratchet

In the previous sections we have discussed the different ways in which high‐energy molecules regulate self‐assembly processes. The different scenarios have been presented in an order of increasing complexity ranging from straightforward templated self‐assembly to the sophisticated kinetic schemes of driven self‐assembly. The examples taken from biology and chemistry have illustrated that a correlation exists between the level of complexity in the energy consumption pathway and the functional complexity of the system. Compared to thermodynamically driven self‐assembly (Class 1), the introduction of an energy dissipation element into the system permitted temporal control over the functions of the self‐assembled material (Classes 2 and 3). The presence of kinetic asymmetry in the energy consumption pathway permitted storage of the energy released from fuel‐to‐waste conversion in a non‐equilibrium high‐energy structure (Class 4). It appears that, if chemists are to harness the full potential of chemical fuel driven self‐assembly, attention should be focused on this final class of systems. For that reason, in this section we explore the chemical origins of kinetic asymmetry in more detail and show that the same kinetic asymmetry drives biomolecular machinery and synthetic molecular machines.

It is important to realize that Equation (2), which provides the composition of the system at constant chemical potential, can be rewritten as Equation 7.

(7) $${\left[\frac{\left(1+\frac{k_{1,\;Wf}}{k_{1,\;Ff}}\right)}{\left(1+\frac{k_{1,\;Wb}}{k_{1,\;Fb}}\right)}\right]}^{2}{\left[\frac{\left(1+\frac{k_{3,\;Wf}}{k_{3,\;Ff}}\right)}{\left(1+\frac{k_{3,\;Wb}}{k_{3,\;Fb}}\right)}\right]}^{-1}=K_{r}$$

This shows that the ratcheting constant, *K*_r_, is exclusively determined by the rate constants of the fuel and waste reactions in steps 1 and 3. Some of the rate constants are apparent first order rate constants that include the fuel and waste concentrations, which are constant under stationary conditions.^[62]^ The potential installation of kinetic asymmetry (*K*_r_≠1) in the system does not at all depend on the equilibrium constants for steps 2 and 4, but exclusively on differences in the transition states of steps 1 and 3. This shows that the ratcheting constant has an exclusively kinetic origin. The occurrence of kinetic asymmetry in the system implies the presence of an information ratchet. In the case of self‐organization, an information ratchet can be defined as a situation in which the capacity of the building block to catalyze the fuel‐to‐waste reaction is different for the unassembled (M) and assembled (M_2_) states.

## 8 Information Ratchets Cause Unidirectional Motion

Information ratchets are well‐known to cause unidirectional motion in biological and synthetic machines.[^149^, ^150^, ^151^, ^152^, ^153^] Unidirectional motion is a non‐equilibrium phenomenon; random Brownian motion can only be overcome at the expense of energy consumption.^[154]^ Therefore, the information ratchet forms a link between chemically fueled unidirectional motion and the self‐assembly of non‐equilibrium molecular systems. Although this Review is dedicated to self‐assembly processes, and although information ratchets have been extensively described within the context of molecular machines, we have nonetheless preferred to discuss chemically fueled unidirectional motion in the same way we have discussed self‐assembly to facilitate recognition of the analogy between directional motion and driven self‐assembly.

### 8.1 Concept

**Figure 14:**
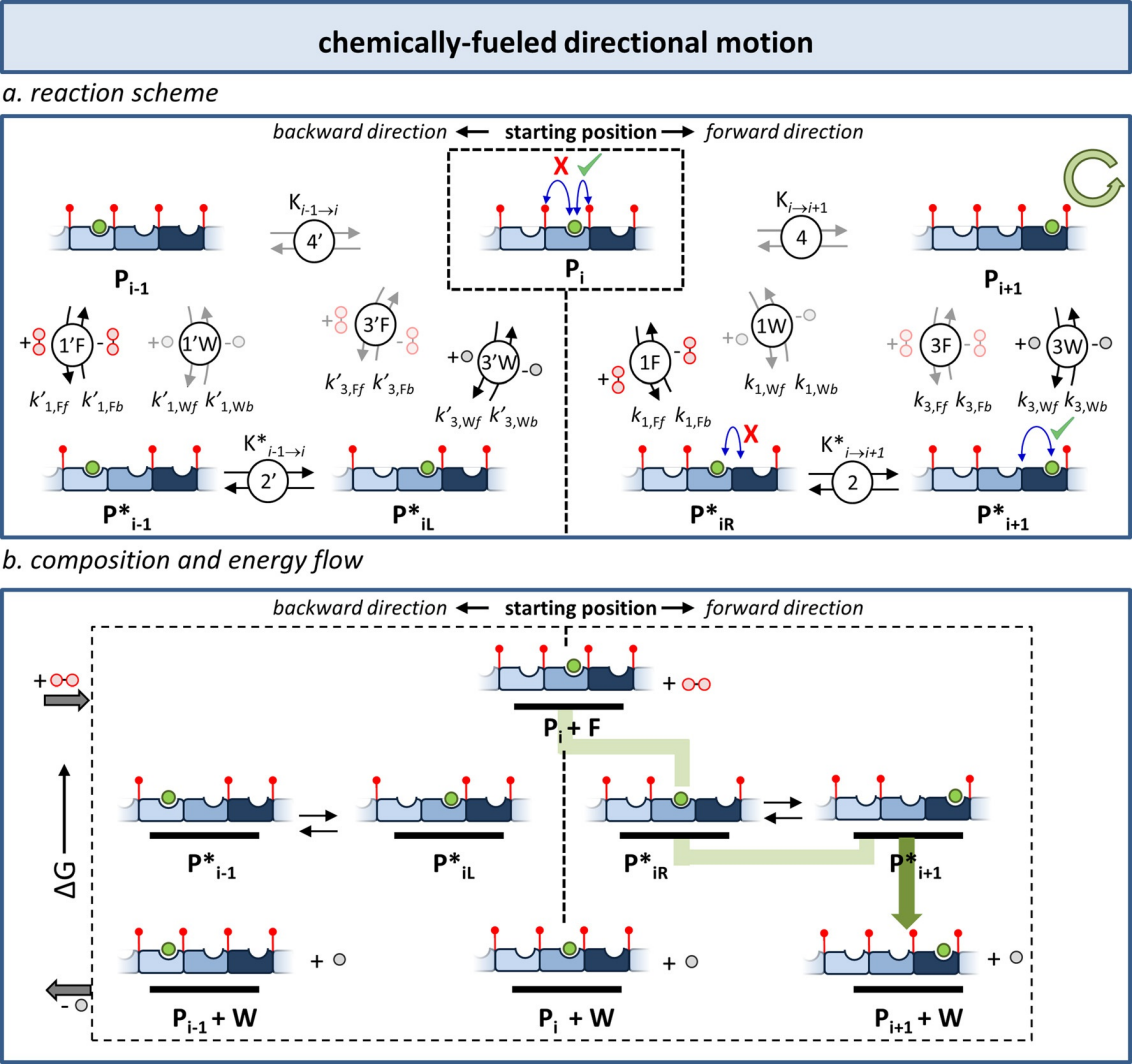
Chemically fueled unidirectional motion. a) Model used to illustrate how kinetic asymmetry leads to unidirectional motion. b) Energy diagram to explain how energy dissipation leads to motion in the forward direction. The green trajectory represents the preferential energy dissipation pathway leading to motion in the forward direction. The dark green arrow represents the step in which waste molecules are generated.

An information ratchet in a molecular machine implies that the position of a moving unit determines the direction of its movement.[^150^, ^151^, ^152^, ^153^] As one possible model to illustrate chemically fueled directional movement, we have taken a linear polymer composed of identical building blocks that contain a binding site for a guest (Figure 14 a). The guest can move along the polymer track by hopping to a neighboring binding site on the left‐ (P_i‐1_) or right‐hand (P_i+1_) side of the original position (P_i_). However, movement of the guest is impeded by the presence of physical barriers between the binding sites. Now, suppose that the presence of a chemical fuel triggers a chemical reaction that causes the removal of the barriers, a process indicated in Figure 14 a with the blue double‐headed arrows. This reaction—marked 1F and 3′F—leads to the transformation of the original structure P_i_ into P*_iR_ or P*_iL_, respectively, depending on whether the barrier to the right‐ or left‐hand side of the guest is removed. In the absence of the barrier, equilibration of the guest between two neighboring binding sites occurs, and—assuming that the binding sites are degenerate—both accessible binding sites will be occupied evenly (P*_iR_ and P*_i+1_ on one side and P*_iL_ and P*_i‐1_ on the other side). Next, a chemically distinct waste‐generating reaction reinstalls the barriers and, thus, restores the original polymer, where movement of the guest is blocked. Depending on which binding site was occupied in the activated structure, this leads to the formation of structures P_i‐1_, P_i_, and P_i+1_. As a result of a single chemical activation–deactivation cycle, the guest is now redistributed over the three binding sites P_i‐1_, P_i_, and P_i+1_.

The question one needs to ask is how such a fuel‐driven cycle can lead to directional motion. Directional movement in the forward direction implies that, after one cycle, more guests have moved to position P_i+1_ compared to position P_i‐1_. It is evident that such a situation can never be achieved for a system that is fully symmetric, because in that case the cycles leading to P_i+1_ and P_i‐1_ are identical and, consequently, after one cycle binding sites P_i‐1_, P_i_, and P_i+1_ must be occupied in a statistically controlled 1:2:1 ratio. Asymmetry is required and in this model system, asymmetry is present in the structure of the building blocks that compose the polymer track. In our model, the structure is such that the binding site for the guest is closer to the barrier on the right‐hand side than to the barrier on the left‐hand side. In cases where the fuel–waste reactions that occur at the barriers are affected by the presence of a guest in the binding site, the asymmetry of the building block implies that the reactions occurring at the right‐ and left‐hand barriers will be affected to different extents by the guest. In Figure 14 a this difference is indicated with the green tick and red cross above the blue double‐headed arrows. It is this “communication” between the occupied binding site and the reactivity at the barriers that creates the conditions for the installation of an information ratchet, which drives directional motion. In our model, the binding of a guest enhances the rate at which the fuel removes the right‐hand barrier (*k*_1,F*f*_≫*k*′_3,F*f*_), causing a more rapid population of P*_iR_ than P*_iL_ (see green trajectory in Figure 14 b). In the absence of the barrier, fast equilibration between sites P*_iR_ and P*_i+1_ occurs. However, the presence of guest in the binding site disfavors the backward reaction that re‐installs the barrier (*k*_1,W*b*_≪*k*_3,W*b*_), which implies the conversion of P*_i+1_ into P_i+1_ occurs faster than the conversion of P*_iR_ back into P_i_. As a consequence of kinetic asymmetry in the cycle, the pathway that moves the guest from P_i_ to P_i+1_ is much faster compared to any of the other possible pathways in the system that would either keep the guest in position P_i_ or move it in the opposite direction to position P_i‐1_. The result of kinetic asymmetry is preferential motion in the forward direction.

In chemically driven self‐assembly, continuous energy supply is required to keep the high‐energy assembly populated.^[62]^ In chemically fueled directional motion, the situation is different because structures P_i_ and P_i+1_ are identical on the molecular level. This difference emerges from an analysis of the energy flow in a single cycle. Fuel activation can potentially lead to four different states, which are degenerate from an energetic point of view. However, as a result of kinetic asymmetry in the activation pathway, the activated structures P*_iR_ and P*_i+1_ are predominantly populated. The presence of kinetic asymmetry in the deactivation pathway then leads to the preferential formation of P_i+1_, thereby liberating waste. At a constant fuel concentration, this process repeats itself continuously and repetitive cycles drive the guest to position P_i+2_, P_i+3_,…, P_i+*n*_.

### 8.2 Biology—Unidirectional Motion of Kinesin

Kinesin 1 is the founding member of the kinesin superfamily of motor proteins that play an important role in many cellular processes, such as intracellular transport, mitosis and meiosis, and control over microtubule dynamics.^[155]^ Kinesin 1 moves stepwise towards the (+)‐end of the microtubule along single protofilament tracks (see Section 6.2). Each step bridges a distance of around 8 nm, which corresponds to the distance between two α/β‐tubulin dimers.^[156]^ The directional movement of kinesin is driven by the conversion of ATP into ADP+P_i_, which proceeds at the expense of one ATP per step.

**Figure 15:**
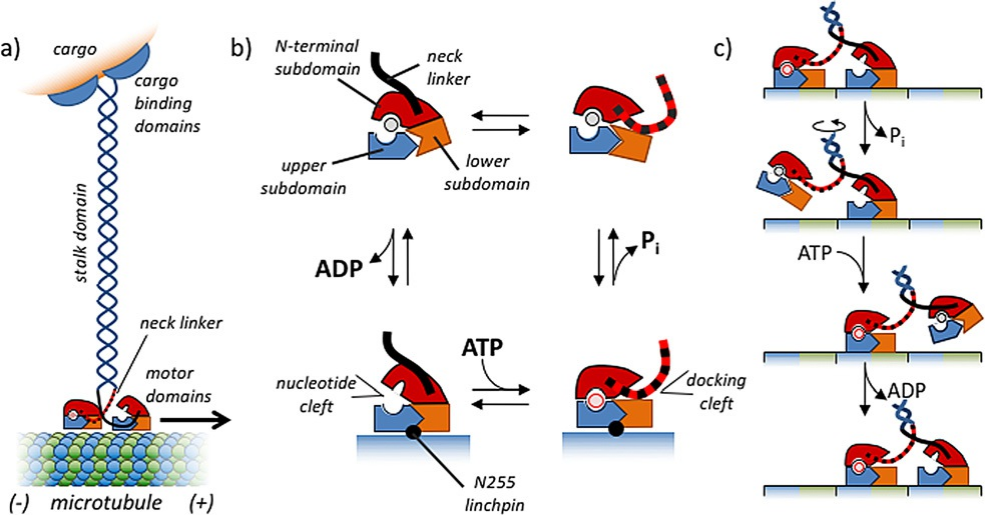
Schematic representation of kinesin 1. b) Structural changes in the motor domain as a function of binding site occupancy by ADP and ATP. c) ATP‐fueled directional motion of kinesin towards the (+)‐end of a microtubule. Figures (b) and (c) are inspired by Ref. [159].

Kinesin 1 is a dimeric protein composed of two identical parts, each with a mass of around 120 kDa (Figure 15 a). Dimerization occurs through the formation of a coiled coil in the stalk domain, which positions two motor domains at one end and the cargo‐binding domains at the opposite end of the structure. A key element of the structure is the neck linker, a segment of 15 amino acids directly involved in ATP‐induced structural rearrangement of the motor domains and the transduction of those changes to the translocation of cargo.

The motor domains are responsible for directional motion, which is a consequence of the structural reorganization of three subdomains in response to ATP binding and hydrolysis, as well as ADP dissociation (Figure 15 b).[^157^, ^158^, ^159^] These structural changes affect three key processes: the binding of the motor domain to the microtubule surface, the exchange of nucleotides in the active site, and the movement of the neck linker. A key role is played by the conserved “linchpin” residue (N255). The interaction of this residue with α‐tubulin leads to the repositioning of the three subdomains. Disengagement of the N‐terminal subdomain and the upper subdomain leads to opening of the so‐called “nucleotide cleft”, which allows dissociation of ADP. At the same time, alignment of the upper and lower subdomains strongly increases the affinity of the motor domain for the microtubule. Subsequent binding of ATP to the active site of the microtubule‐bound motor domain provides energy to organize the motor domain into a new strained conformation. Through a seesaw motion, the N‐terminal subdomain reconnects to the upper subdomain, which closes the nucleotide cleft, but also opens up a docking cleft for the neck linker, which subsequently binds in the direction of the microtubule (+)‐end. This is a key step in the process, as it is the moment where repositioning of the neck linker leads to directional movement of cargo along the microtubule. At the same time, the rearrangement leads to formation of the catalytic pocket and ATP is rapidly hydrolyzed into ADP and P_i._ Following ATP hydrolysis, P_i_ dissociates but ADP remains trapped in the motor domain, as the nucleotide cleft remains closed. However, ADP is not able to maintain the motor domain in the strained conformation. The interaction between the upper and lower subdomains weakens and, as a consequence, the motor domain loses its affinity for the microtubule surface and dissociates from the neck linker bound in the docking cleft.

The question here, is how these processes combine to allow directional movement of kinesin 1 along the tubulin tracks. As a starting point, to describe a single step of kinesin 1 on the microtubule surface, we take the state where both motor domains are strongly bound to the microtubule (Figure 15 c).^[159]^ Strong affinity is assured by the presence of ATP in the trailing head and the absence of nucleotide in the leading head. The neck linker of the trailing head is constrained in the docking cleft. Following the hydrolysis of ATP, dissociation of the trailing head from the microtubule takes places, but ADP remains trapped in the binding pocket and the neck linker remains positioned in the docking cleft. At this point, binding of ATP by the leading head promotes a forward step of the trailing head by docking the neck linker of the leading head. This movement also leads to release of the strained neck linker of the trailing head from the docking cleft. Attachment of the—former—trailing head to the forward tubulin causes the dissociation of ADP, which closes the cycle. The astonishing perfection of this machine is evident from its performance: kinesin hydrolyzes ATP at a rate of around 100 molecules per second.^[160]^ Considering the step size of 8 nm per molecule of ATP, this implies that kinesin moves along a microtubule at a speed of around 800 nm s^−1^!

The directional movement of kinesin 1 is a sophisticated process in which a series of structural reorganizations follow each other in a highly orchestrated sequential manner. However, as previously shown in a very elegant manner by Astumian,^[152]^ abstraction of the process by focusing on the kinetic scheme makes it apparent that kinetic asymmetry is the basis of directional movement. The dimeric nature of kinesin 1 is essential, as it creates an asymmetry between the two microtubule‐bound motor domains. The chemically distinct nature of the trailing and leading heads permits the installation of an information ratchet in which the binding and conversion of fuel is dependent on the position of the motor domain. Indeed, biochemical analysis shows that ATP hydrolysis occurs exclusively when the motor domain is in the backward position, but that the release of waste (ADP) occurs selectively from the forward position. The coupling of the activation and deactivation reactions to the relative position of the motor domains installs the information ratchet that drives unidirectional motion.

### 8.3 Chemistry—Unidirectional Motion in a Nanomachine

In recognition of the fact that the biological machinery composed of protein‐based motors and pumps is essential for maintaining the cell in a non‐equilibrium state, chemists have been fervently dedicated to the development of artificial molecular machines for applications in materials science, nanotechnology, and drug‐delivery systems. Over the past decades, enormous progress has been made and intricate molecular systems have been reported that indeed show directional motion. However, most of these systems use light as an energy source, whereas molecular machines that exploit chemical energy are only recently emerging.

**Figure 16:**
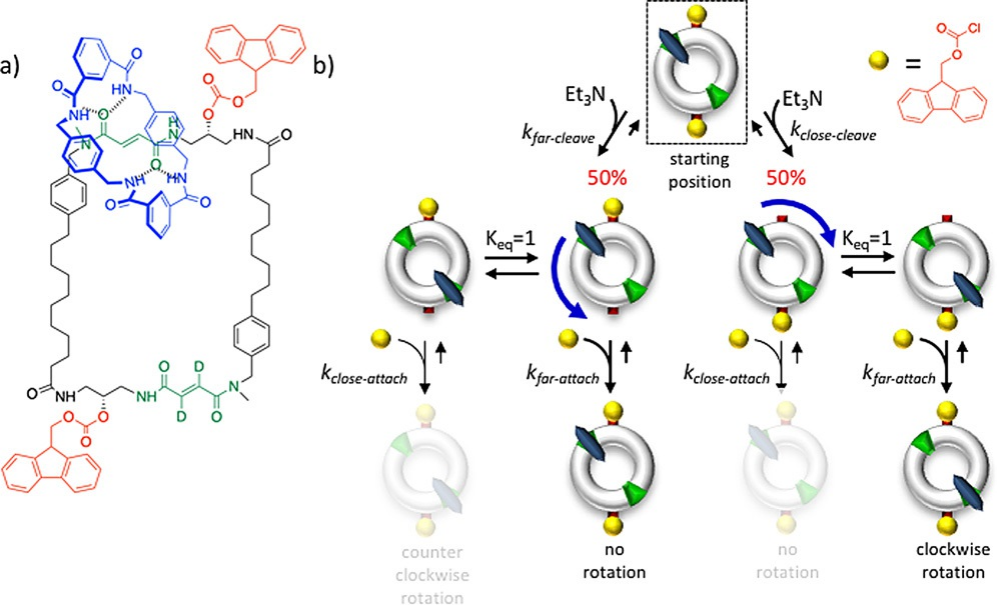
a) Chemical structure of a [2]catenane used for Fmoc‐Cl‐fueled directional rotation of the small ring (blue) around the large ring. b) Illustration of how a single removal–attachment cycle leads to net rotation of the blue ring in a clockwise direction.

A very elegant demonstration of how chemical fuels can be used to drive directional motion using an information ratchet has been provided by Leigh and co‐workers (Figure 16).^[48]^ They prepared a [2]catenane featuring two mechanically interlocked rings of different sizes. In a comparable role as the protofilaments of microtubules, the large ring serves as a cyclic track for the movement of the smaller ring. The small ring can shuttle between two binding sites on the track, but the passage is blocked by the presence of two bulky stoppers—fluorenylmethoxycarbonyl groups—that are attached in proximity to the binding sites. Removal of the bulky stoppers in the presence of base eliminates the barriers to movement and the small ring can equilibrate between the two degenerate binding sites. Importantly, the bulky stoppers can be re‐introduced by a chemical reaction with Fmoc‐Cl, which reinstates the barriers to movement. This reaction is orthogonal to the cleavage reaction, which implies that experimental conditions can be created in which the [2]catenane is subject to continuous cleavage and attachment of the stoppers as long as chemical fuel—Fmoc‐Cl—is available.

Directional motion requires the installation of an information ratchet, which implies that the rate of the forward and/or backward reactions must be sensitive to the position of the moving component on the track. In the [2]catenane, such a situation occurs in the attachment reaction that (re‐)introduces the stoppers on the large ring. The bulky size of Fmoc‐Cl renders this reaction more difficult when the neighboring binding site is occupied by the small ring. The decrease in rate relative to the same reaction next to the unbound binding site is around fivefold (*k*_far‐attach_>*k*_close‐attach_). On the other hand, the rate of the cleavage reaction is not sensitive to the presence of the small ring (*k*_far‐cleave_=*k*_close‐cleave_), because this reaction is mediated by the triethylamine‐triggered abstraction of a proton from the fluorenyl methine group that is five bonds remote from the attachment point.

Starting from the [2]catenane containing the stoppers, it can be illustrated how a single cleavage–attachment cycle causes directional movement of the small ring (Figure 16 b). Considering that the cleavage reaction is insensitive to the presence of the ring, removal of the top and bottom stoppers occurs at the same rate. In both cases removal of the barrier permits movement of the ring to the other binding site through a counterclockwise or clockwise movement. The binding sites are degenerate and, consequently, the small ring will occupy these sites equally. However, since subsequent re‐introduction of the stoppers occurs more rapidly at the anchoring points that are more remote from the ring, the equilibrium structures are not evenly converted back into the [2]catenane with two stoppers. For the clockwise pathway, the major product is the [2]catenane containing the ring in the opposite position, whereas for the counterclockwise pathway, the [2]catenane with the ring in the original position dominates. Thus, after a single fuel–waste cycle the ring has either moved in a clockwise direction to the other position or has remained in the original position. Net cycling in the clockwise direction continues as long as Fmoc‐Cl is present in the system. Evidently, the efficiency and complexity of this system is still considerably far from that of kinesin 1. The calculated rate is around 12 h for each 360° rotation and a large quantity of fuel is consumed without generating motion, because the cleavage pathway is not selective. Nonetheless, this system provides a fundamental contribution to understanding how information ratchets can be implemented in chemically fueled molecular machines.

## 9 The Design of Information Ratchets

The discussions on driven self‐assembly processes and unidirectional motion illustrate that information ratchets are essential to maintain a chemical system away from equilibrium.^[62]^ It is, therefore, important to take a closer look at the chemical origin of the information ratchet. Taking as reference the conceptual scheme used to introduce driven self‐assembly, it is important to recognize that the reactions [Eq. 8]:

(8) $$M+F\overset{k{}_{1,Ff}}{\underset{k{}_{1,Fb}}{↔}}M{}^{*}\overset{k{}_{1,Wb}}{\underset{k{}_{1,Wf}}{↔}}M+W$$

on the left‐hand monomer side of the scheme, and [Eq. 9]

(9) $$M{}_{2}+F\overset{k{}_{3,Ff}}{\underset{k{}_{3,Fb}}{↔}}M{}^{*}{}_{2}\overset{k{}_{3,Wb}}{\underset{k{}_{3,Wf}}{↔}}M{}_{2}+W$$

on the right‐hand assembly side are identical to the sequence of events that describe how an enzyme (E) converts substrate (S) into product (P) through the intermediate complex ES [Eq. 10].

(10) $$E+S\overset{k{}_{1}}{\underset{k{}_{-1}}{↔}}\mathrm{ES}\overset{k{}_{2}}{\underset{k{}_{-2}}{↔}}E+P$$

The importance of this observation, which has been previously pointed out by Astumian,[^63^, ^152^] is that the self‐assembling building blocks involved in non‐equilibrium chemistry must be catalysts for the conversion of fuel into waste.^[161]^ However, for the installation of kinetic asymmetry, it is of crucial importance that the catalytic parameters of the building block, that is, the Michaelis–Menten parameters *K*_M_ and *k*_cat_, are affected by the self‐assembly process. Kinetic asymmetry requires that fuel is bound preferentially by the unassembled monomer M—rather than by the assembly M_2_—but that catalysis is more efficient in the activated assembly M*_2_ compared to the activated monomer M*. This is precisely what happens in microtubule formation (Section 6.2). GDP–GTP exchange is only possible when tubulin is in the unassembled form. Exchange does not occur when tubulin is embedded in the microtubule lattice. On the other hand, free tubulin is not catalytically active. The catalytic site is only formed when tubulin associates to form the microtubule.

It is important to realize that kinetic asymmetry is an essential condition to populate the high‐energy assembly M_2_, but not sufficient by itself. Population of M_2_ also requires that the fuel‐to‐waste conversion steps 1 and 3 occur faster than the equilibration steps 2 and 4. In other words, M_2_ must be populated at a higher rate than the rate at which it disassembles into 2 M. The presence of a kinetic barrier to disassembly of the high‐energy state is a second essential requirement.

**Figure 17:**
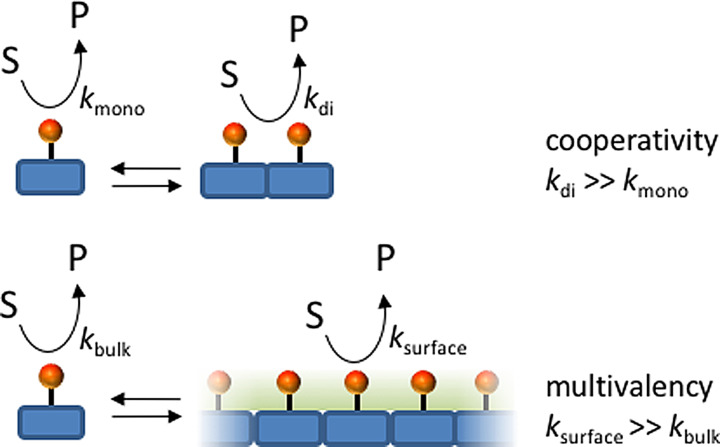
Exploitation of cooperativity and multivalency for the installation of kinetic asymmetry.

These insights provide a valuable piece of information for the design of chemically fueled driven self‐assembly processes. Building blocks should be selected for their capacity to catalyze the conversion of fuel into waste. It is worth pointing out that this does not necessarily need to occur through noncovalent interactions. Transient covalent activation of the building block is equally possible, in analogy with covalent enzyme catalysis.^[162]^ The activated building block should, of course, have the tendency to self‐assemble in a thermodynamically controlled fashion, but, importantly, self‐assembly should be accompanied by activation of the catalysis. Whereas catalyst activation in nature is extensively regulated by conformational changes in the enzyme, chemists can achieve the same goal by using cooperative catalysis^[163]^ and multivalency^[164]^ (Figure 17). Cooperative catalysis implies that the catalytic site is composed of multiple catalytic units. This can become a powerful tool if self‐assembly can be used to bring those functional groups in proximity. Recently, the first examples have been described in which a chemical fuel templates the formation of an assembly and, in doing so, creates the conditions for its own destruction.[^165^, ^166^] On the other hand, the exploitation of multivalency to install catalytic activity relies on changes in the local chemical environment (pH, polarity, ionic strength…) near the surface of a multivalent assembly. This may favorably affect the reaction rates or even open up different mechanistic pathways for fuel‐to‐waste conversion.^[167]^

From an experimental point of view, a study of the properties of the high‐energy assembly M_2_ would strongly benefit from the development of experimental set ups that allow the installation of a non‐equilibrium steady state under continuous fuel consumption. An important step in this direction was reported by Hermans and co‐workers, who developed a membrane reactor that permitted continuous exchange of fuel and waste, but not the building blocks.^[131]^

## 10 Outlook

We have described how chemical fuels are used to control structural organization in both biology and chemistry. We have identified different scenarios, which have been presented in an order of increasing complexity and, accordingly, with increased functionality. The comparison between examples from biology and chemistry demonstrates the wide gap between the natural and synthetic worlds, in particular related to the exploitation of chemical fuels to drive systems away from equilibrium. Evidently, this gap finds justification in the fact that chemists have only recently started to explore synthetic chemically fueled self‐assembly processes with the precise purpose of creating artificial non‐equilibrium systems. This initial phase is characterized by exploration, discovery, an increased understanding of the involved mechanisms, and a logical discussion of key definitions. Indeed, the precise meaning of non‐equilibrium self‐assembly is a matter of debate. We would like to add to the debate the following reflections, which emerge from the conceptual treatments presented in this Review.

Equilibrium on the molecular level implies that the rate of the reaction in the forward direction equals the rate of the same reaction in the reverse direction. A situation of non‐equilibrium implies a disparity between these rates; detailed balance is broken. Such a situation can be established in two ways that, however, have a fundamentally different origin and for that reason it is creating confusion. A transient non‐equilibrium situation is created when the concentration of one of the species involved in the reaction is changed (for example, the fuel concentration). This leads to a transiently higher reaction rate in the forward reaction until the systems evolves to the new equilibrium state. This is what happens in any chemical reaction after mixing of the reactants. This is the basis of templated self‐assembly under dissipative conditions (Class 2) and dissipative self‐assembly (Class 3) when fuel is added batchwise. However, we have discussed that, in these cases, the composition of the two equilibria (that is, between M and M_2_ (4) and between M* and M*_2_ (2)) never changes as a consequence of energy dissipation. The relative ratio of free and assembled building blocks corresponds always to the composition at thermodynamic equilibrium. Two thought experiments can be used to illustrate that the existence of a non‐equilibrium state in these cases is indeed strictly related to variations in the fuel concentration. Firstly, if one could imagine that at some moment in time all the fuel and waste reactions were instantly turned off, this would not lead to any shift in the concentrations of any species, because their ratios are entirely dictated by the equilibrium constants *K*_4_ and *K*_2_. Secondly, if systems from Class 2 or 3 would be studied at constant fuel and waste concentrations, this would lead to a composition of the system that is entirely dictated by the relative thermodynamic stabilities of the systems’ components. Under these conditions, all separate reaction steps would have identical rates for the forward and reverse directions. The most populated species in the system would be M*_2_, which is the thermodynamically most stable species in the system. Systems belonging to Classes 2 and 3 can never develop a stationary non‐equilibrium state for the self‐assembly processes (equilibria 2 and 4).

From a non‐equilibrium point of view, an entirely different situation is created when kinetic asymmetry in the energy dissipation pathway is present (Class 4). Likewise, in this case, a stationary state is obtained under constant fuel and waste concentrations, but with a key difference in that detailed balance for equilibria 2 and 4 is broken. In other words, under stationary conditions, persistent currents are present in the system, which indicates that a stationary non‐equilibrium state is installed for the self‐assembly processes. This implies that only in this case do chemical fuels drive the formation of a truly dissipative structure (M_2_), according to the definition of Prigogine.^[3]^ It marks the only case in which energy consumption is truly required to maintain structural order.

We would like to point out that this distinction is not an intent to value one class over the other. As illustrated by the examples from both biology and chemistry, all systems have their own specific utility. Indeed, it is the entire ensemble of kinetically controlled processes that leads to the emergent properties of a cell. From the origin‐of‐life perspective, a clear connection emerges with the concept of dynamic kinetic stability advanced by Pross and co‐workers as being a characteristic feature of life.[^5^, ^168^, ^169^] It appears evident, though, that in terms of the development of synthetic systems with emergent properties, chemical fuel driven self‐assembly (Class 4) is potentially the most rewarding case. It represents the only class in which chemical energy is transferred to a self‐assembly process and stored in a high‐energy organized state. It is, therefore, worth reflecting on the potential gain by focusing experimental attention on the formation of high‐energy assemblies, such as M_2_, rather than thermodynamically stable assemblies, such as M*_2_. Microtubules use stored energy to do mechanical work by generating pushing and pulling forces. In vitro studies have shown that a single depolymerizing microtubule can generate about ten times the force developed by a motor protein.^[170]^ Dynamic instability of microtubules, which is a property that derives from the accumulation of lattice strain, permits a search and capture of kinetochores—which are specialized anchoring sites on the chromosomes—during cell division at a rate that would not be possible for a thermodynamically stable polymer.^[171]^ In other words, the storage of energy can lead to improved properties. In this context it is worth adding that kinetic asymmetry is not limited to the molecular level, but can also emerge on the macroscopic level through spatially controlled energy delivery. For example, we have recently shown that a hydrogel containing catalytic nanoparticles can be maintained in a stationary non‐equilibrium state upon local irradiation with UV light.^[172]^ Enhanced catalytic activity was observed under non‐equilibrium conditions as a result of persistent concentration gradients in the gel. These observations indicate that the installation of kinetic asymmetry is an important tool that can be used to create non‐equilibrium systems. Energy storage in non‐equilibrium systems leads to enhanced properties and this provides a powerful incentive to focus efforts on the development of synthetic self‐assembly processes that display kinetic asymmetry in their energy dissipation pathways.

## Conflict of interest

The authors declare no conflict of interest.

## Biographical Information

*Krishnendu Das completed his B.Sc and M.Sc in Chemistry at the University of Calcutta, India. In 2017, he obtained his Ph.D. from the Indian Association for the Cultivation of Science, Kolkata, India. After spending one year as a postdoctoral researcher at the Indian Institute of Science Education and Research, Kolkata, India, he moved to Italy to join the group of Leonard Prins at the University of Padova as a Marie Curie Seal‐of‐Excellence@UNIPD fellow to work on the dissipative self‐assembly of chemical nanoreactors. His research interests include systems chemistry and non‐equilibrium supramolecular systems*.

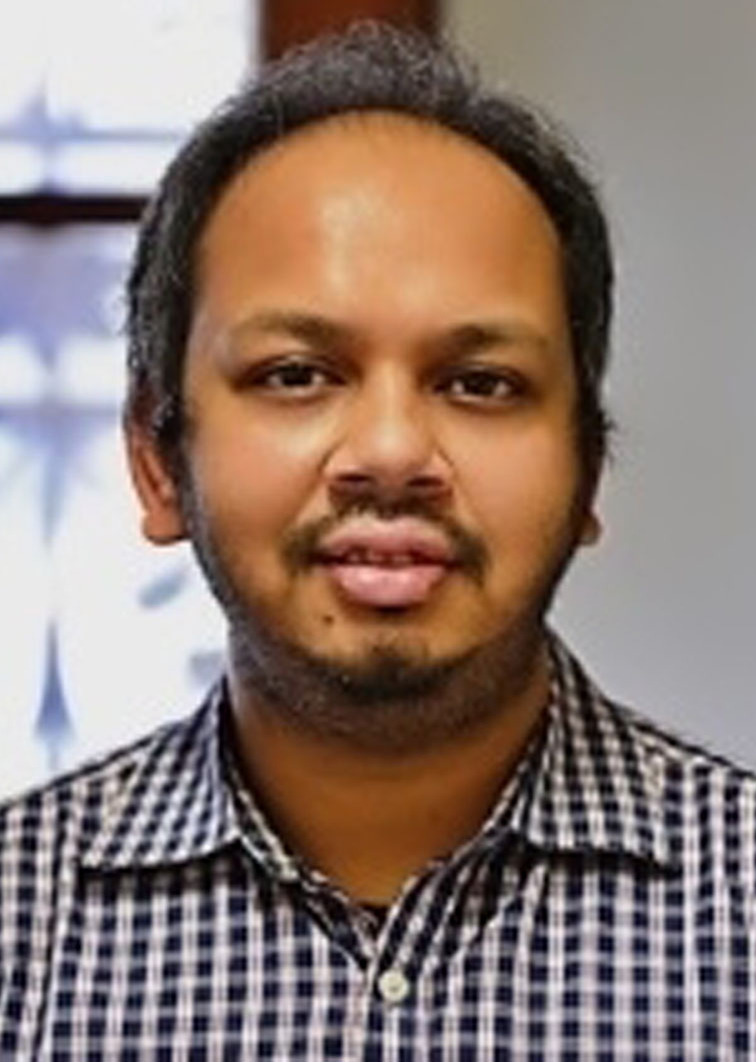

## Biographical Information

*Luca Gabrielli received his PhD from the University of Milano‐Bicocca (2013), under the supervision of Prof L. Cipolla. After postdoctoral research in the groups of Prof. F. Mancin (University of Padova, 2014) and Prof. C. A. Hunter (University of Cambridge, 2017), where he was awarded with an IF‐MCSA fellowship, he then moved back to the University of Padova (2019), as a tenure‐track assistant professor*.

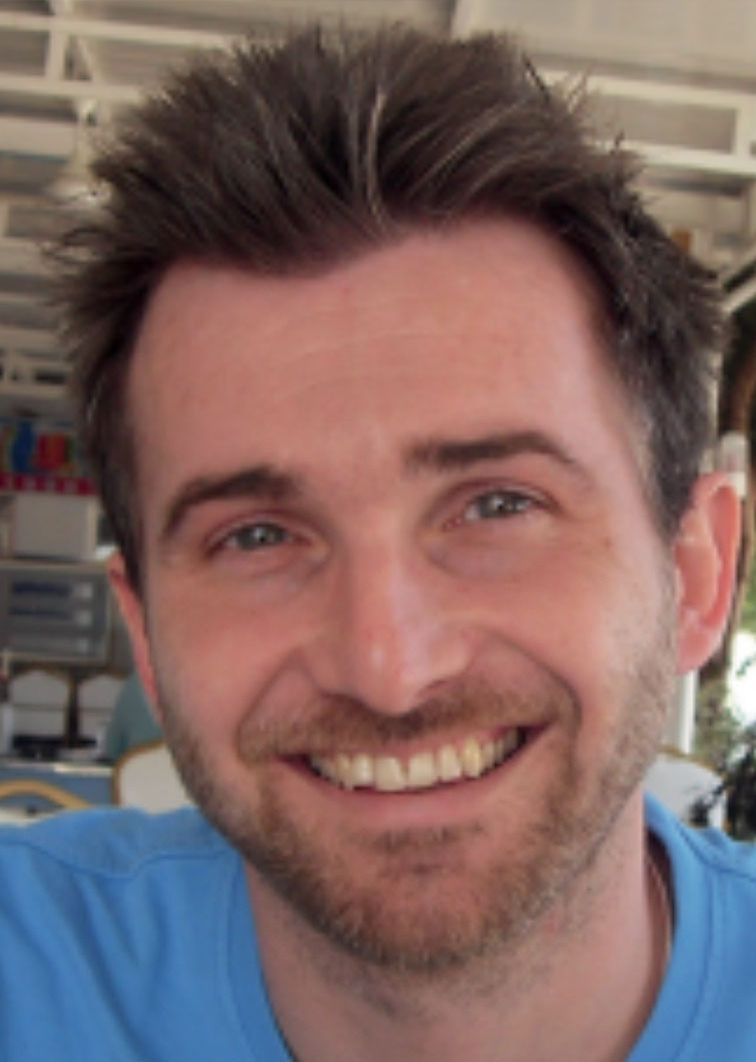

## Biographical Information

*Leonard Prins (1974) is full professor in organic chemistry at the University of Padova, Italy. In 2001 he obtained his PhD from the University of Twente, Netherlands, after which he carried out postdoctoral research at Caltech, Pasadena (CA), and the University of Padova. His research concerns systems chemistry with a particular focus on non‐equilibrium self‐assembly and catalysis*.